# Monitoring ecological recovery of reclaimed wellsites: Protocols for quantifying recovery on forested lands

**DOI:** 10.1016/j.mex.2019.03.031

**Published:** 2019-04-04

**Authors:** Anne C.S. McIntosh, Bonnie Drozdowski, Dani Degenhardt, Chris B. Powter, Christina C. Small, John Begg, Dan Farr, Arnold Janz, Randi C. Lupardus, Delinda Ryerson, Jim Schieck

**Affiliations:** a University of Alberta, Augustana Campus, 4901 46 Ave., Camrose, AB T4V 2R3, Canada; b InnoTech Alberta Inc. Edmonton, AB, T6N 1E4, Canada; c Canadian Forest Service, Edmonton, AB, T6H 3S5, Canada; d Enviro Q&A Services, Edmonton, AB, T6K 0S1, Canada; e Teck Resources Ltd., 421 Pine Ave., Sparwood, BC, V0B 2G0, Canada; f Alberta Environment and Sustainable Resource Development, Edmonton, AB, Canada; g Alberta Environment and Parks, Environmental Monitoring & Science Division, Edmonton, AB, T5J 5C6, Canada; h Eco-Logical Consulting Ltd., Gibbons, Alberta, T0A 1N0, Canada

**Keywords:** Monitoring protocol, Ecological recovery, Reclaimed wellsites, Soil sampling, Vegetation sampling, Boreal forest

## Abstract

We developed a scientifically robust and financially sustainable monitoring protocol to enable a consistent assessment of ecological recovery of physical, chemical, and biological indicators at certified reclaimed industrial wellsites in forested lands in noutheastern Alberta. Using the developed protocols, data can be generated from measurement of soil, vegetation, and landscape indicators at reclaimed wellsites and adjacent reference sites. We selected the appropriate vegetation, soil, and habitat indicators for a long-term reclamation monitoring program and have provided sampling protocols for the selected indicators here. The protocols may be used to identify and prioritize indicators of reduced ecosystem health and to track ecological recovery of reclaimed sites over time. The development of these integrated monitoring protocols is a first step towards successful and consistent long-term monitoring to assess ecological recovery of certified wellsites in Alberta. These protocols can be applied to wellsites and other similar sized disturbances in other forested regions too.

## Graphical abstract

**Figure:**
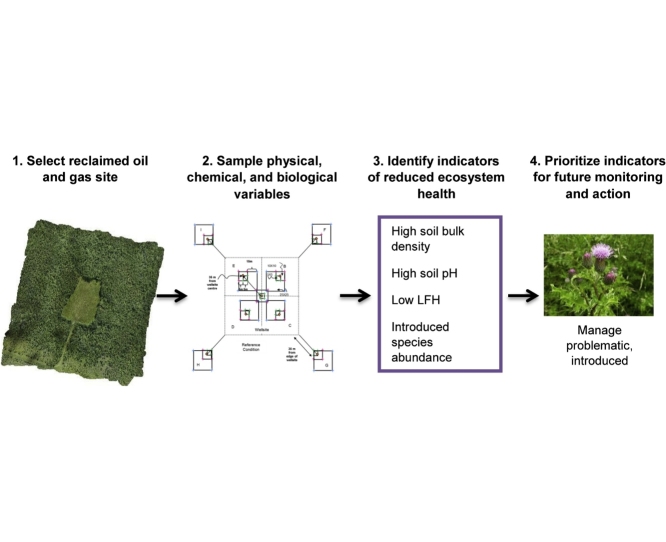

**Specifications Table**

**Table:** 

Subject Area:	*Agricultural and Biological*
More specific subject area:	*Reclamation*
Protocol name:	*Sampling soil and vegetation on well sites*
Reagents/tools:	*Not applicable*
Experimental design:	*The sampling design samples two different areas within a single assessment unit (called the Monitoring Site): the wellsite, and a reference area (i.e., a paired comparison design)*^1^*. For the purposes of this protocol, the wellsite is restricted to the disturbance footprint of the well pad. The reference area, selected so as not to have a footprint of human disturbance, is the reference against which ecological recovery is assessed.* *The design minimizes the effects of spatial variability of the monitoring site by systematically selecting sampling points – this increases the ability to precisely measure temporal change in selected indicators. The ease of use and the sampling efficiency makes it a better choice than random sampling for this monitoring program*.
Trial registration:	*Not applicable*
Ethics:	*Not applicable*

**Value of the Protocol**

**Table:** 

• *Protocol is easy to follow and understand*. • *Can be used to evaluate ecological recovery of vegetation, soil, and habitat indicators*. • *Can be used for consistent long-term reclamation monitoring*.

## Description of protocol

Forested Land sites in Alberta are currently subject to the 2010 Reclamation Criteria for Wellsites and Associated Facilities for Forested Lands [1]. However, prior to development of this protocol, there was no way to evaluate ecological recovery of reclaimed wellsites after they were reclaimed. This document provides monitoring protocols for quantifying ecological recovery of vegetation, soil, and habitat indicators on certified reclaimed forested lands^2^ (adapted from [2,3]). Given the level of detail we have provided in this protocol document, we think that any oil and natural gas reclamation scientist would find this protocol useful and easy to follow; the components that a given research team may be interested in studying may vary and by splitting out the protocol into various components (e.g., downed wood, vegetation, soils) the researchers can readily study the components of interest to their given study. Additionally, we built our protocols from sampling protocols used on thousands of sites across Alberta [4].

### Monitoring program design

The sampling design and protocols sample two different areas within a single assessment unit (called the Monitoring Site): the wellsite, and a reference area (i.e., a paired comparison design)^3^ . For the purposes of this protocol, the wellsite is restricted to the disturbance footprint of the well pad. The reference area, selected so as not to have a footprint of human disturbance, is the reference against which ecological recovery is assessed.

The design minimizes the effects of spatial variability of the monitoring site by systematically selecting sampling points – this increases the ability to precisely measure temporal change in selected indicators. The ease of use and the sampling efficiency makes it a better choice than random sampling for this monitoring program.

## Monitoring site selection

The goal of the early stages of Program implementation was to expand on the range of key site characteristics (Appendix A, Table 5) represented in the monitoring database developed as part of the Pilot Program. As the program progresses sites can be selected to build in replication of selected key site characteristics to add statistical power to data analysis and to improve representation in the region.

In addition, specific monitoring sites may be worth revisiting periodically (perhaps every 5 or 10 years) to monitor trends in key monitoring parameters – protocols for determining which monitoring sites to revisit will be developed as more data are gathered.

### Site selection methodology

The following steps were used to select a site for monitoring in Alberta – sampling in other locations can select alternative means for prioritizing site selection:

1 Obtain list of potential sites from Alberta Energy Regulator (AER) and Alberta Environment and Parks (AEP) databases for the region(s) to be sampled in a given year.
2 Determine Candidate Site Ratings from Appendix A, Table 5 and identify the highest rated candidate sites^4^ .
3 Review available data in AER, AEP and AbaData^5^ records to further help screen sites.
4 Identify final list of candidate sites and any lower-priority sites in the area of a candidate site that could be sampled if time permits.
5 Confirm landowner approval to sample and request current status of site (e.g., active grazing) (see Section Securing Landowner Permission Protocol for more details).
6 Conduct a reconnaissance trip to the candidate site to make sure the site is suitable for inclusion in the Program. The site may be rejected permanently if clearly not reclaimed or another disturbance is present. Site logistics issues such as access are also assessed at this time – this is particularly important for forested land sites as often it turns out that a road is washed out, or has been decommissioned, resulting in challenges in accessing sites and selection of alternate sites.
7 Implement monitoring protocols on remaining sites.

Site selection should focus on sites that have higher ratings. Sites with lower ratings can be added to the Program where they are in proximity (short distance or travel time) to higher-rated sites^6^ – this will help expand the range of monitoring sites while maximizing Program efficiency.

### Site records

The review of records provides information to help classify sites for future analysis and to help explain the monitoring results.

In addition to the information in Appendix A, Table 5, records that should be captured (where available) include:

1 Reclamation certificate application form.
2 Reclamation certificate assessment data (e.g., Detailed Site Assessment, Phase I).
3 Comments by the Reclamation Inspector and landowner at the inquiry^11^
4 Spill and remediation records (potentially found on the Environmental Site Assessment Repository – http://aep.alberta.ca/lands-forests/land-industrial/programs-and-services/environmental-site-assessment-repository.aspx).
5 Complaint records (and any work required to address the complaint).
6 Whether or not the wellsite was deemed to be a potential problem site [5] and the resulting adjustment to the site liability value.

These records may be found in databases of the Alberta Energy Regulator and Alberta Environment and Parks – some may be electronic and some may require access to paper archives.

## Plot establishment protocols

Plot establishment is designed to facilitate field sampling by having; landowner permission to enter the land; a predetermined route to site centre recorded on an access sheet; an estimated timeframe for getting to the site; and, knowledge of potential access hazards.

Several tools are available for developing the predetermined site access route. Oil Trax and Avenza PDF maps were used in the Pilot Program. The latter was the best but it requires that a modeler prepare the maps and import wellsite coordinates into an app-specific map.

;1;

Accessing monitoring sites has multiple components:

• Prior to the first site visit map/GIS and data reconnaissance work in the office that gathers as much data as possible about accessing the site and the site history are needed to assist field crews in their first visit to the site.
  ○ The wellsite centre should be labeled and GPS coordinates from the map/GIS recorded for the wellsite centre and four corners on Datasheet #1 (Appendix B).
  ○ The need for surveying for ground disturbance needs to be established prior to the first visit to the site. In Alberta, this involves setting up an account on Alberta OneCall (http://www.albertaonecall.com/) and submitting ground disturbance requests a minimum of 3 business days before sampling is going to be conducted. Companies with potential below-ground pipelines etc. should contact you to let you know whether or not there is a conflict and whether marking of lines will be required (if you haven’t heard back then you may need to check the site to see if it has been marked).
• Finally before going into the field, additional maps and descriptions are prepared and put together into a site information package that can be used to aid in locating the site, and access materials are compiled to facilitate data collection during future monitoring visits.
• During the first visit to the monitoring site, the most efficient route is found, and potential hazards are described on Datasheet #1 (Appendix B) and supplied maps.
  • Ensure that compass declination is set appropriately for the location. Declination for the region is determined by checking on the GPS and recorded on Datasheet #1 (Appendix B). The accuracy of the GPS used during site establishment is also recorded on Datasheet #1.
  • Where site access is complicated, record the GPS locations of turnoffs, corners, significant landmarks, and parking locations. Include detailed direction and distance measures to aid staff in relocating all access points and site centre. This will be most relevant for locations after you have turned off a main road/highway.

### Securing landowner permission protocol

The majority of forested land sites are on public land in the Green Area – therefore permission from the public land manager and occupants is required to access and sample land. It is also important to notify the company responsible for the Forest Management Agreement where the Monitoring Site is located. Note: it is important to tell the occupant that work is being conducted on behalf of the government – the contact is good relations but the occupant can’t refuse access for government-sponsored research.

#### Private land access

Several counties and municipal districts have land ownership maps that will provide a starting point for current contact information. Depending on the time since certification, the certificate application and transmittal letter will also contain landowner information that may be current.

There are some key points to remember when accessing and working on private land:

• No materials can be left on site: no flagging, rebar, or equipment at all will be left at the site, and crews will be diligent to not leave any garbage of any kind on site.
• It is critical to the program that crews be very respectful of land owners as ambassadors for the program. This includes:
  ○ No quadding on private property at all unless specifically requested by landowners.
  ○ Take corporate logos off the vehicle (or cover them up) while on private property.
  ○ If you find gates open, leave them open. If you find gates closed, close them.

### Plot layout

**Field Equipment Needed:**

• Cell phone for communications (be prepared that, depending on location, phones may not always work – satellite phones may be an alternative for very remote sites)
• 2-way radios for communications among partners
• Clipboard
• Site maps and wellsite information package
• GPS and compass
• 9 (1 per 10 × 10 m plot – centre location gets metal marker) – permanent magnetic metal markers per site
• 135 pigtails to mark the nested 5 × 5 m and 10 × 10 m plots, downed woody debris (DWD) transect start points, quadrant corners, and wellsite centre within the wellsite and reference areas
• 4 – 50 m tapes, 4–100 m tapes and 4–30 m tapes
• Multiple colors of flagging tape e (e.g., brown = DWD, pink = 10 × 10 m, orange = 5 × 5 m – could match up with colors on the cheat sheet – see Appendix A)
• Fine tipped coloured marker (to delineate polygons on human disturbance sketch)
• Pencils for recording data on datasheets
• Pin locator – magnetic metal detector
• Plot layout cheat sheet (see Appendix A)
• Datasheets #1 to 3

#### Wellsite

**Fig. 1:**
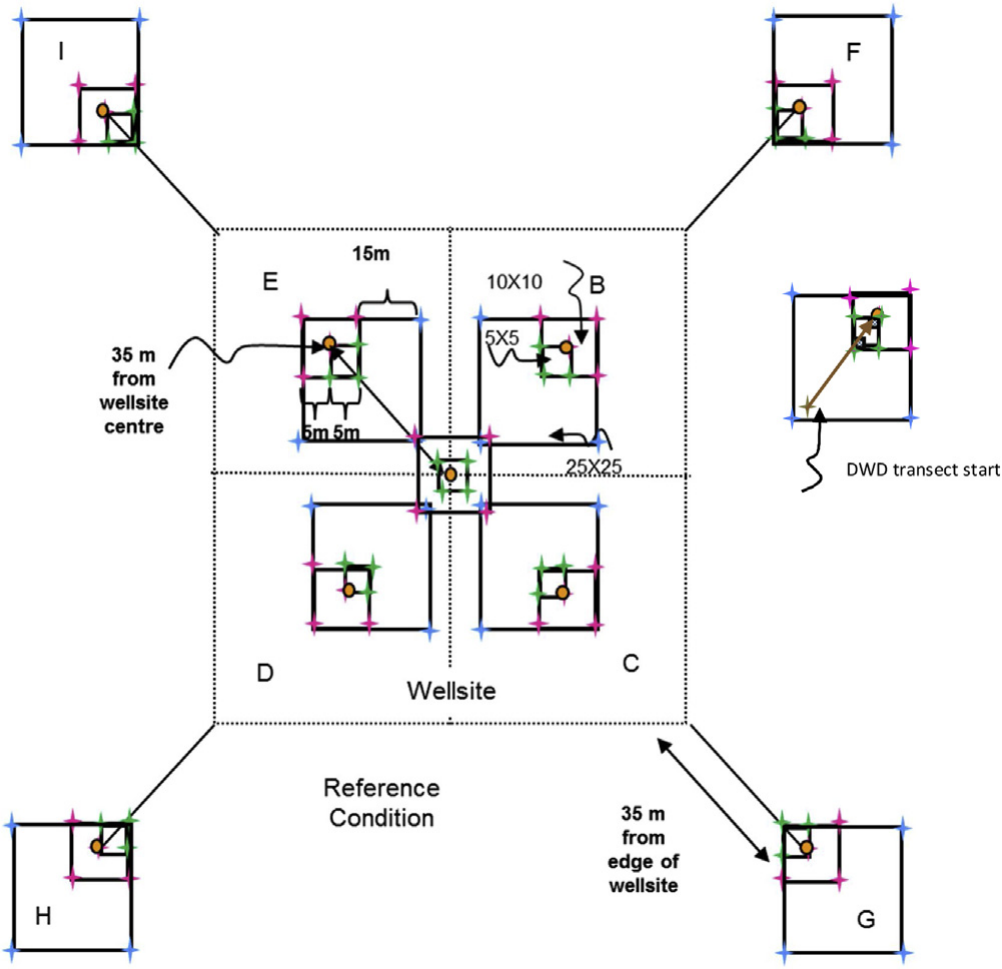
Sampling layout of wellsites and adjacent reference areas. Nested square plots and pigtail placement for the 5 × 5 m (green), 10 × 10 m (pink), and 25 × 25 m (blue) plots, which are located within the four quadrants of the wellsite, and the area surrounding each of the reference area plots, which are also referred to as a quadrant. Pigtail placements for plots including in the 25 × 25 m square plot that includes the DWD transect. Note: plots are not drawn to exact scale.

For level and near-level sites, the following sampling design will be used (Fig. 1). On monitoring sites where there is significant across-slope curvature, it is important that all slope elements are represented. Hence the sampling squares should encompass all slope positions within the 1 ha site with one square in each convergent-divergent sequence across the slope and this should be noted on the site disturbance sketch.

Every effort should be made to reduce the impact of the plot layout and sampling work (e.g., trampling, weed movement, damage to property such as fences and gates).

;1;

**Procedures:**

• When the field crew arrives onsite, the first step is to identify the wellsite centre, which will be the centre point for the reclamation wellsite 1 ha plot too. It must be located as precisely as possible using a hand-held GPS with an accuracy of < 7 m (GPS coordinates will have been identified from the maps and GIS investigation prior to the site visit). If due to poor satellite coverage accuracy values from the GPS are > 7 m, this is noted on Datasheet #2 (Appendix B). A metal detector can also be used to identify the wellsite centre.
• At wellsite centre place a pigtail in the ground and flag it so that you can readily identify the wellsite centre. Note that you may have trouble identifying the wellsite centre so you may have to measure the diagonals between the four corners and then identify the wellsite centre as the point where the two diagonal lines intersect.
  ○ A permanent metal marker (or metal magnet) will be inserted in the sample hole at wellsite centre after the soil sampling is complete so that the location can be readily identified with a metal detector during future visits to the site.
  ○ Note that these permanent markers will also be used on private land, but approval for them should be obtained from the landowner. Record the GPS coordinates at the wellsite centre on Datasheet #2 (Appendix B).
• The crew will lay out four sub-ordinal transects that are oriented to the four corners of the wellsite (e.g., if the wellsite is square in cardinal directions, then the bearings of the 4 transects would be northeast 45°, southeast 135°, southwest 225°, northwest 315° – if not cardinal then adapt the directions of the four transects to angles so they intersect the four corners of the wellsite). Each quadrant is assigned a letter code (wellsite = B, C, D, E; reference = F, G, H, I – see Fig. 1).
  • Record the bearings for the Wellsite Corners for B, C, D, E quadrants on Datasheet #2 (Appendix B) and also record the GPS coordinates for the centre of each 10 × 10 m plot (i.e., 10 GPS measurements per site including wellsite centre and well bore^7^).
• Establish the first transect for the wellsite – it is most efficient to have both crew members establish each transect together and use the plot layout cheat sheet (Appendix A). Leaving the tape on the ground is useful for the next steps. Carry an extra 50-m tape and 30-m tape and 17 pigtails with you. Using a 100-m tape attached to the wellsite centre pigtail, lay out your tape along the bearing of the sub-ordinal transect. You should flag the different plots and quadrant corners with different colors of flagging to help identify them (e.g., brown = DWD, blue = 25 × 25 m, pink = 10 × 10 m, green = 5 × 5 m). Hint: it is helpful to use 2 people and triangulate with a single tape (e.g., 50 m) to complete the final 2 corners for the 5 × 5 m, 10 × 10 m, and 25 × 25 m plots. You may prefer to use GPS coordinates to identify and label the 50 × 50 m plot corners (note that there is a bit of room for error in the locations of the 50 × 50 m quadrants because during the plant censuses the observer usually will not travel all the way to the edges of the quadrant).
  • When you have laid out 3.5 m of tape insert a pigtail (this will be the pigtail for the corner of the centre 5 × 5 m plot).
  • When you have laid out 6.7 m of tape insert a pigtail (this will be the pigtail for the near diagonal corner of your 25 × 25 m transect; note – if you do not have trees ≥ 25 cm DBH you don’t have to insert this pigtail).
  • When you have laid out 7.1 m of tape insert a pigtail (this will be the pigtail for the corner of the centre 10 × 10 m plot for soil sampling).
  • When you have laid out 10 m of tape, insert a pigtail – this will be the start point for the coarse woody debris (CWD) DWD transects.
  • When you have laid out 25 m of tape, insert a pigtail – this will be the start point for the small woody debris (SWD) DWD transects.
  • Continue laying out the tape measure until you reach 27.9 m from wellsite centre and insert a pigtail (this will be the near corner of your 10 × 10 m plot).
  • Continue out to 35 m from the wellsite centre and insert a pigtail (this is the centre of your 10 × 10 m plot). Record the GPS coordinates on Datasheet #2 (Appendix B).
  • Continue to 42.1 m (this will be the far diagonal corner for the 10 × 10 m and 25 × 25 m plots).
  • Insert pigtails for the remaining sides of the 10 × 10 m (and 25 × 25 m plots if appropriate) by measuring 10 m and 25 m (using the 30-m tape), N or S and E or W (depending on the quadrant of the wellsite you are setting up). If there are not a lot of trees in the way you can triangulate to identify the other two corners of each square plot.
  • Add two additional pigtails for the remaining sides of the 5 × 5 m plots by measuring 5 m, N or S and E or W (again will depend on the quadrant) using the 30-m tape.
  • Finally continue measuring the tape out from the far end of the 10 × 10 m plot (located at 42.1 m from the wellsite centre) to the edge of the wellsite or to a distance of 70.7 m (whichever comes first):
    ○
      ○ if the wellsite corner is less than 70.7 m (this will apply if the wellsite is < 1 ha) record the distance from wellsite centre on Datasheet #2 and insert pigtail, or
      ○ if the edge of the wellsite is beyond 70.7 m from the plot centre then place the wellsite quadrant corner pigtail at 70.7 m but still run the tape out to the edge of the wellsite and record the distance to the edge of the wellsite on Datasheet #2 (Appendix B).

Repeat the procedures described above for the remaining sub-ordinal transects that have not yet been established.

All flagging and pigtails must be removed after each visit, but magnetic metal markers should be inserted along the transect at the plot centre of each 10 × 10 m plot so the plots can be re-identified in future visits to the site.

;1;

#### Selecting adjacent reference areas

The following section describes establishment procedures for adjacent reference areas located 35 m from the four wellsite corners.

If one or more of the reference areas selected by this method are not representative of the recovery target for the wellsite (e.g., a wetland vs. upland target, or a different ecosite phase) then:

• Try to find another location for the reference area(s) near the wellsite;
• If that fails see Section Selecting Adjacent Reference Areas below for non-adjacent reference area procedures.

Adjust the location of the reference area if necessary to ensure the location is undisturbed (e.g., not on a pipeline or access road).

;1;

To establish adjacent reference area plots, walk to the corner of the wellsite footprint and then roll out the 100-m tape and lay out the line transect at the same bearing as for the same sub-ordinal quadrant transect.

• Insert a pigtail at 10 m (this will be the start of the coarse woody debris (CWD) DWD transect).
• Insert a pigtail at 25 m – this will be the start point for the small woody debris (SWD) DWD transects.
• Insert a pigtail at 27.9 m (this will be the near corner of the 10 × 10 m plot).
• Insert a pigtail at 35 m (this will be the centre of the 10 × 10 m plot and the far corner of the 5 × 5 m plot and the end of the DWD transect).
• Insert a pigtail at 42.1 m (this will mark the far corner for the 10 × 10 m reference plot).
• Insert a pigtail at 63.3 m (this will be the far diagonal corner of the 25 × 25 m plot – only do this if you have trees ≥ 25 cm DBH present).
• Insert pigtails for the remaining sides of the 10 × 10 m (and 25 × 25 m if trees ≥ 25 cm DBH are present) plots by measuring 10 m (or 25 m for the 25 × 25 m plots), N or S and E or W (depending on the wellsite or reference site quadrant).
• Add two additional pigtails for the remaining sides of the 5 × 5 m plots by measuring 5 m, N or S and E or W (depending on the quadrant). See Fig. 1 for diagram of pigtail layout.
• Insert a pigtail at 70.7 m and then add 2 additional pigtails for the remaining sides of the quadrant (which will be used for the plant census). *If the wellsite is < 1 ha (i.e., the distance to corner of quadrant is < 70.7 m) then adjust the length of the reference transect to the length of the diagonal distance for the wellsite (i.e., the wellsite and reference areas should have the same area sampled for vascular plant surveys).

#### Selecting non-adjacent reference areas

When the land adjacent to the wellsite is not suitable as a reference area then there will have to be an alternative strategy to locate reference areas. This will require an expert in the field identifying an area as close as possible to the wellsite that is undisturbed and representative of the natural conditions that were likely to be present on the wellsite prior to disturbance.

A total reference area that is similar in size to the wellsite (1 ha) should be sampled – following modified protocols that adapt the protocols described throughout the document to the shape of the reference condition site. GPS points should be marked for the centres of the 10 × 10 m plots that are sampled in the reference area plots.

## Site description protocols

A variety of information about a site should be captured in the Program records to allow for: improved data analysis and reporting; updating the Program protocols; and, future research. The information is obtained through reviews of existing records and through site observations.

### Site observations

Sketches and photographs provide a permanent record of the site as of the date the monitoring was conducted. This will be particularly helpful in case a site is selected for later reassessment. Effective sketches and photographs can also be used to visually link monitoring findings to the site which may provide insights into patterns that raw data will not provide.

#### Site sketch

Draw sketches of the wellsite and each of the reference areas – these can be combined if the reference areas are adjacent to the wellsite but may have to be separate sketches if the reference areas are at some distance. Sketches should represent both historical information culled from records (e.g., well bore and access road locations) and from onsite observations.

Sketches will include:

• North arrow to orient site
• Wellsite development information (e.g., wellhead, access road and sump location)
• Location of nearby roads, including old logging roads
• Presence and/or evidence of standing water
• Arrows to indicate slope direction
• Bare soil areas
• Excessive weed areas
• Erosional and depositional areas
• Sample locations, plots and transects (based on the Plot Layout Protocols in section 3)
• Datasheets #3A and #3B (Appendix B)

Use the datasheets provided to complete a map outlining all disturbance evidence present at the site (e.g., wellhead bore location, roads nearby) and the reference areas. Write the type of disturbance in the polygons using the codes described under “Human Disturbance” included on the datasheets. Once mapping is completed, review the diagram to ensure that it reflects the site conditions.

#### Site photographs

**Field Equipment Needed:**

• Digital camera with a 35 mm focal length and a quality setting of at least 3 Mega-pixels (take extra batteries and charger)
• Backpack (or some other suitable object) for scale
• Datasheet #4 (Appendix B)

**Procedure:**

• Use “landscape” orientation for all photos.
• Take six photographs at each wellsite (record the photo numbers on Datasheet #4):
  ○ Four Transect Photos – Standing at wellsite centre take a photograph at eye level in each of the four sub-ordinal directions so that you are pointing towards the transect associated with each Quadrant (B, C, D, E – begin with ‘B’ quadrant and move clockwise).
  ○ Canopy Photo – Standing at wellsite center, directly over the pigtail, take a photograph of the canopy looking skyward.
  ○ Representative Site Photo – From anywhere within the 1 ha wellsite take a single photograph that best represents the physical and vegetation characteristics; provide the location and direction of this photo on the site diagram.
• Take five photographs of the reference areas – one of each 10 × 10 m plot that best represents the physical and vegetation characteristics plus a canopy photo. Record which plot you took each photo in on Datasheet #4 (Appendix B).
• In each photo, include a back pack approximately 5 m from the camera for scale.
• Check the resolution and quality of all photos at the site; re-take if the photo is blurry.
• Transfer photo files onto a laptop computer once back at camp or in the office and label them as follows:
  • Transect photos are labeled [Region]_[year]_[site]_“W” or “R”_[quadrant].jpg (e.g., DMG _2013_3_W_C.jpg).
  • Representative site photo for the wellsite is labeled with [Representative] at the end of the label name.
  • All canopy photos have _canopy added to the end of their names.
• Copy all photos to an external hard drive/flash key for backup.

#### Field notes

Field notes should be written while on site. Notes should be recorded on rite-in-rain type of paper using a pencil. Write on one side of the paper only.

Documentation of the personnel involved and procedural issues that arose provides additional context for the data and can assist in future revisions to the Program. Examples of the types of notes to be taken include:

• Date and time of day
• Weather
• Mistakes made
• Changes required to the protocols
• Samples lost or damaged
• Comments on site accessibility and changes to route of travel
• Personnel names and associated roles

Scientifically-defensible, replicated data form the basis for the assessment of the status of ecological recovery for each site. However, there is considerable value in subjective field observations as an additional tool to help explain and validate the monitoring results. Of particular interest are obvious differences between the wellsite and the reference areas.

Examples of subjective observations that can be recorded include:

• General impressions of the monitoring site (e.g., easy to spot wellsite or not)
• Evidence of new disturbances (e.g., ATV tracks, etc.)
• Soil horizon features in reference areas (based on the LFH assessment in section 5.2 and the soil cores in section 5.3), such as cumulative thickness of mineral and organic topsoil horizons (LFH, Ah, Ae, Ahe), upper subsoil features (genetic horizon codes, structure, consistence, properties of mottles), slope positions – information that can be used to understand the soil and landscape context
• Difficulty/ease of digging soil (e.g., compacted, rocky, wet)
• Uniformity of vegetation and soils
• Vegetation health and vigour
• Evidence of invasive plants (weeds) and potential location of ingress (i.e., from adjacent disturbances, etc.)
• Sensory information (e.g., specific sights, sounds, smells)
• Evidence of grazing/trampling
• Evidence of wildlife use (e.g., browse, scat, bedding, travel)

In addition to the observations of the assessor, any comments by landowners, land managers or occupants who may be present at the time of the assessment or that are made during discussions about site access should be recorded.

## Soil assessment

This Section describes the field-based protocols for sampling of soil parameters. Soil sampling should be conducted in the 10 × 10 m plots only after all other sampling has been done at the sites to minimize the effects of the destructive sampling on the other measured indicators. Most of the lab analysis that will then be conducted on the samples is not described in detail in these protocols.

Soil measures include:

• Bulk density – because it has tremendous influence on the soil’s capability for water partitioning, air exchange and plant growth.
• Soil organic carbon – because it is an important indicator of a soil’s ability to sustain plant growth, rooting, water partition and air exchange.
• Soil electrical conductivity (EC) and pH – two useful indicators of soil quality and its capacity to support plant growth. EC in particular is a good indicator of salinity as well as admixing of the surface soil and sub-soil.
• Total Nitrogen (TN) – as it is used to calculate C:N ratios.

### Sampling

Offset the location of any of the soil sampling sites by approximately 1 m if they coincide with the location of the well bore.

;1;

#### Number of samples

**Fig. 2:**
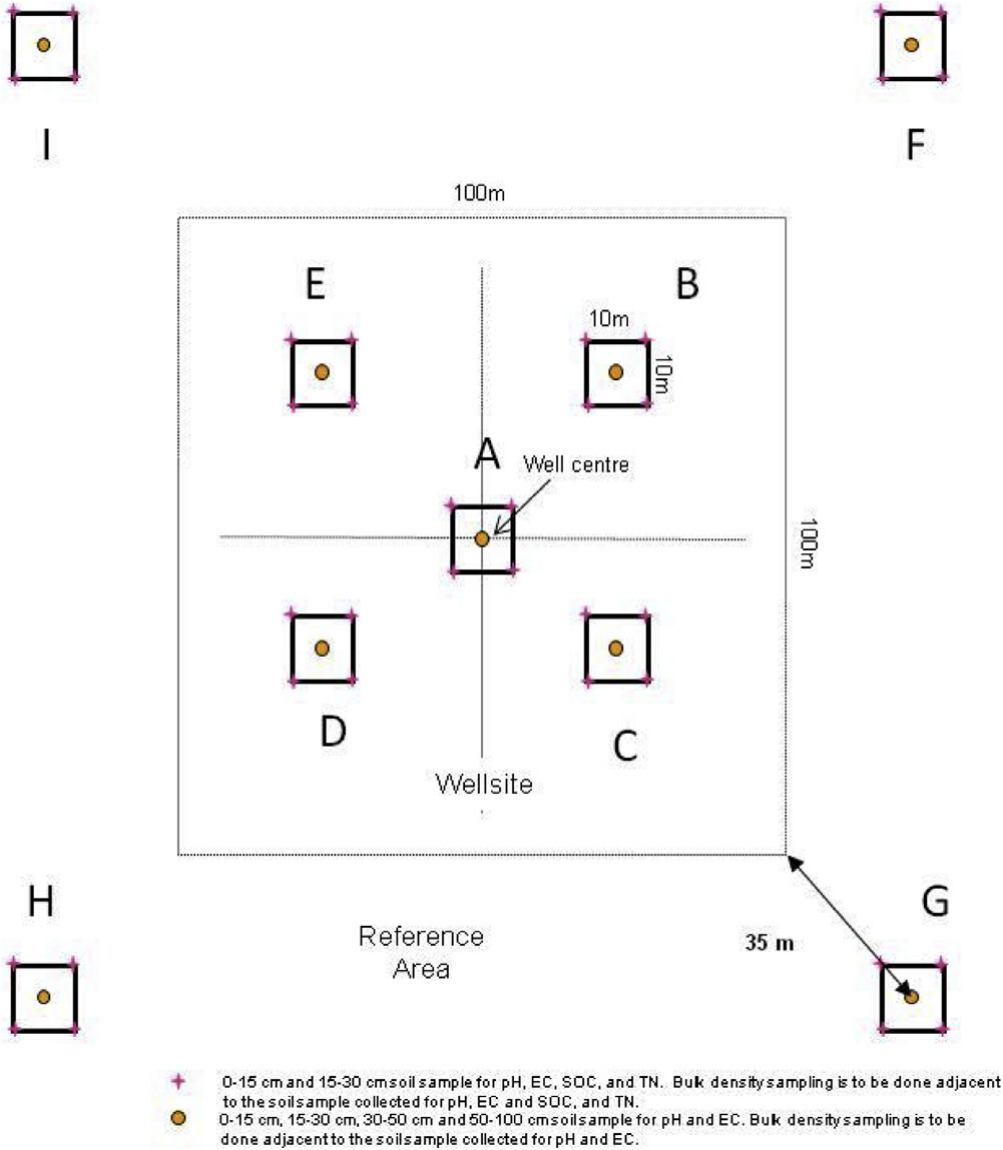
Soil parameters are sampled within the 10 × 10 m plots identified in the diagram.

**Fig. 3:**
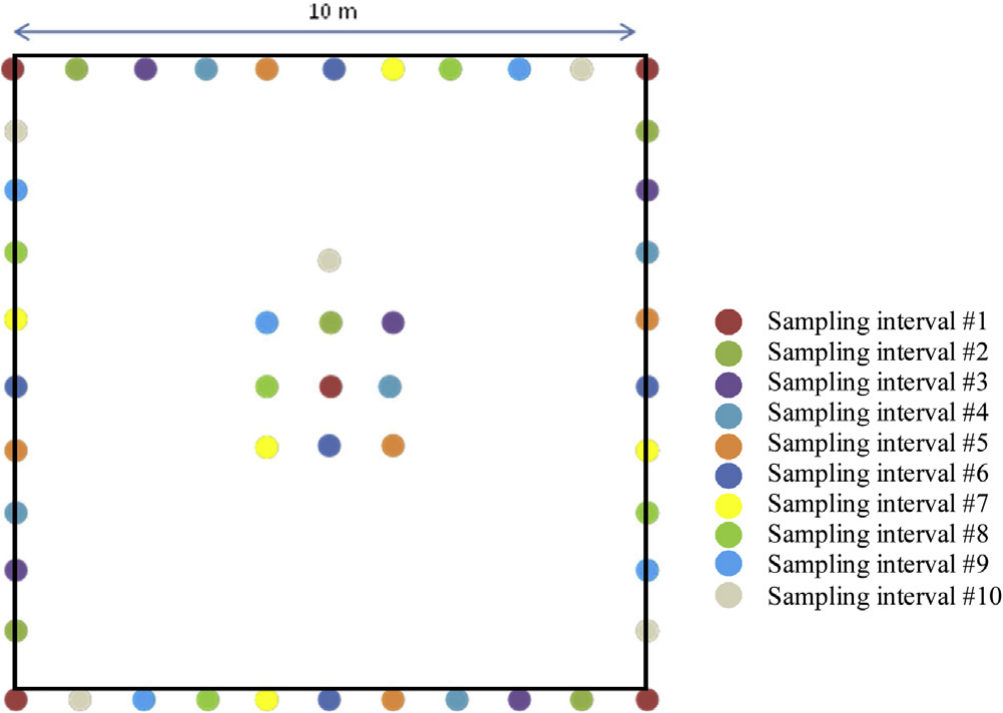
Sampling layout for repeat sampling within each 10 × 10 m plot on the wellsite and reference sites. Each colour represents a different sampling interval, for a total of 10 sampling intervals.

One composite sample per depth made up of 5 cores from each of the 10 × 10 m plots is sufficient for each indicator analysis with the exception of bulk density (Fig. 2, Fig. 3)^8^ . Compositing samples should not be conducted in the field. Samples should be stored separately and composited in the laboratory after bulk density has been measured and the samples have been air-dried and ground to 2 mm.

For soil bulk density measurements, it is suggested on the first initial sampling interval to collect 5 core samples for two depths (0–15 cm and 15–30 cm).

#### Depth of sampling

The sample depth combinations were selected based on the indicator chosen. Two sample depths are recommended: 0–15 cm (0″ to 6″) and 15–30 cm (6″ to 12″), for soil EC, pH, SOC, TN and bulk density. EC and pH will also be monitored at the 30–60 cm (12″ to 20″) and 60–100 cm (20″ to 40″) depths for the centre sampling point in each of the 10 × 10 m plots (Fig. 2).

#### Locations for repeat sampling

It is recommended that the sampling frequency for the soil parameters be 10 years or more depending on the parameter, budget and number of sites. The sampling frequency has not yet been determined and will be determined in a future version of the protocol. There are 10 different sets of sampling locations identified so that soils can be destructively sampled 10 times within each 10 × 10 m plot (Fig. 3). Each sampling point will be located a minimum of 1 m apart from the previous sampling location.

### LFH depth

Organic matter is defined as the LFH layer of the soil horizon. Determining the LFH horizon is usually straight forward in most soil conditions. The organic layer is typically dark in colour, coarse and fibrous (containing rooting systems) whereas the mineral soil is typically lighter in colour, finely particulate, and lacking most roots. LFH does not include live vegetation on the surface.

**Field Equipment Needed:**

• Trowel
• Ruler or tape measure (measured to the scale of mm)
• Datasheet #5 (Appendix B)

**Procedure:**

The thickness of the organic layer is measured at each of the five sampling points within each 10 × 10 m square plot where the soil core is collected from.

Gently insert the trowel into the organic layer and distinguish the transition between the organic layer and the underlying mineral soil.

After distinguishing the transition from LFH to mineral horizon, measure the LFH to the nearest mm and record on Datasheet #5 (Appendix B).

### Bulk density

There are a variety of soil sampling techniques to assess bulk density; the appropriate sampling method depends largely on the distribution of coarse fragments (particles with diameter > 2 mm) at the given site. The most common method is the core method, and should be used when coarse fragments occupy less than 25% by volume [6].

#### Core method

**Fig. 4:**
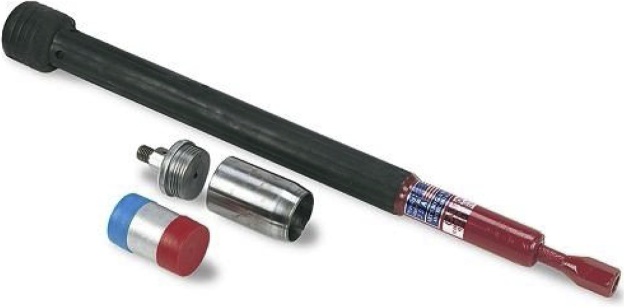
AMS Inc. double-cylinder, drop-hammer soil core sampler.

A double-cylinder, drop-hammer sampler with a liner core is designed to collect an undisturbed soil sample (Fig. 4). The sampler head contains an inner cylinder with a liner and is driven into the soil with blows from a drop hammer. The liner containing an undisturbed soil core can then be removed and, where necessary, trimmed to the end with a knife to yield a core whose volume can easily be calculated from its length and diameter. The weight of this soil core is then determined after drying in an oven at 105°C for 24 h.

**Field Equipment Needed:**

• Double-cylinder core sampler. The most common core diameter range from 2″ to 3″ (5.1 cm to 7.6 cm). Having a second core sampler on hand in case one breaks is recommended
• Two crescent wrenches to tighten the core parts while in the field if they become loose (note that these should be checked regularly – for example, after each sample is collected, as they regularly become loose and this will weaken them and lead to them breaking)
• Clean, dry and uniform stainless steel liners with a known internal diameter and height for volume calculation
• Soil knife or metal spatula
• Polyethylene plastic bags (2 per sample – 7 pound)
• Shipping tag labels (pre-labeled) – insert between the two 7 pound plastic bags
• Pam cooking spray
• Tape measure (to determine core hole depth)
• 2 buckets with lids – it is useful to have a couple of buckets per field crew to help with storage of samples as they are being collected
• Hand pruners for trimming roots from bulk density cores (not used in the Pilot Program)
• Datasheet #5 (Appendix B)

**Lab Equipment Needed:**

• Analytical balance
• Drying oven capable of heating up to 105°C

**Procedure:**

*Lab (pre-sampling)*

• Label shipping tags with appropriate label (naming convention is currently the following: Region-Site Number – Wellsite(W) or Reference (R) – Quadrant (A–I) – Starting depth of sample (0, 15, 30, 60 – e.g., DMG-5-W-C-30) (this should be done in the laboratory before the samples are obtained).

*Field*

• Select a smooth and relatively undisturbed surface at the appropriate sampling point.
• Remove the live vegetation at the surface of the grassland so that the core is collecting the soil rather than live vegetation (e.g., a quick kick of the vegetation with your boot).
• Drive or press the core sampler into the soil sufficiently to fill the inner liner without inducing compaction. In frictional or dense soils, lubricant (e.g., Pam spray) may be required to prevent compaction of the soil and to facilitate emptying the collected core sample from the sampler. When the corer is at the required depth, gently rock in a circular motion to break the contact of the soil core with the ground.
• Carefully remove the undisturbed soil core and trim the ends flush with the edge of the cylinder if necessary (most often the soil breaks off naturally). Resample adjacent to the original sampling point if large coarse fragments or roots protrude from the sample (smaller roots may be trimmed using hand pruners). Any deviation from the original sampling scheme will be recorded by the field staff.
• Be sure to measure the start and end depths using a tape measure to record the length of the core on Datasheet #5 (Appendix B).
• Store the sample in the pre-labelled in polyethylene bags. Tie the bag closed. Store in large durable plastic bag for transport.
• Repeat a second time in the same hole to collect the 15–30 cm depth sample.

*Lab (post-sampling)*:

• Place the sample in an oven set to 105 °C for 24 h.
• Record the weight of the dry soil.

### Chemistry

Soil organic carbon, TN, EC and pH can be analyzed from the same composite sample. The section below describes the sampling protocol for collecting the sample in the field as well as the sample handling, processing and compositing/bulking in the lab.

**Fig. 5:**
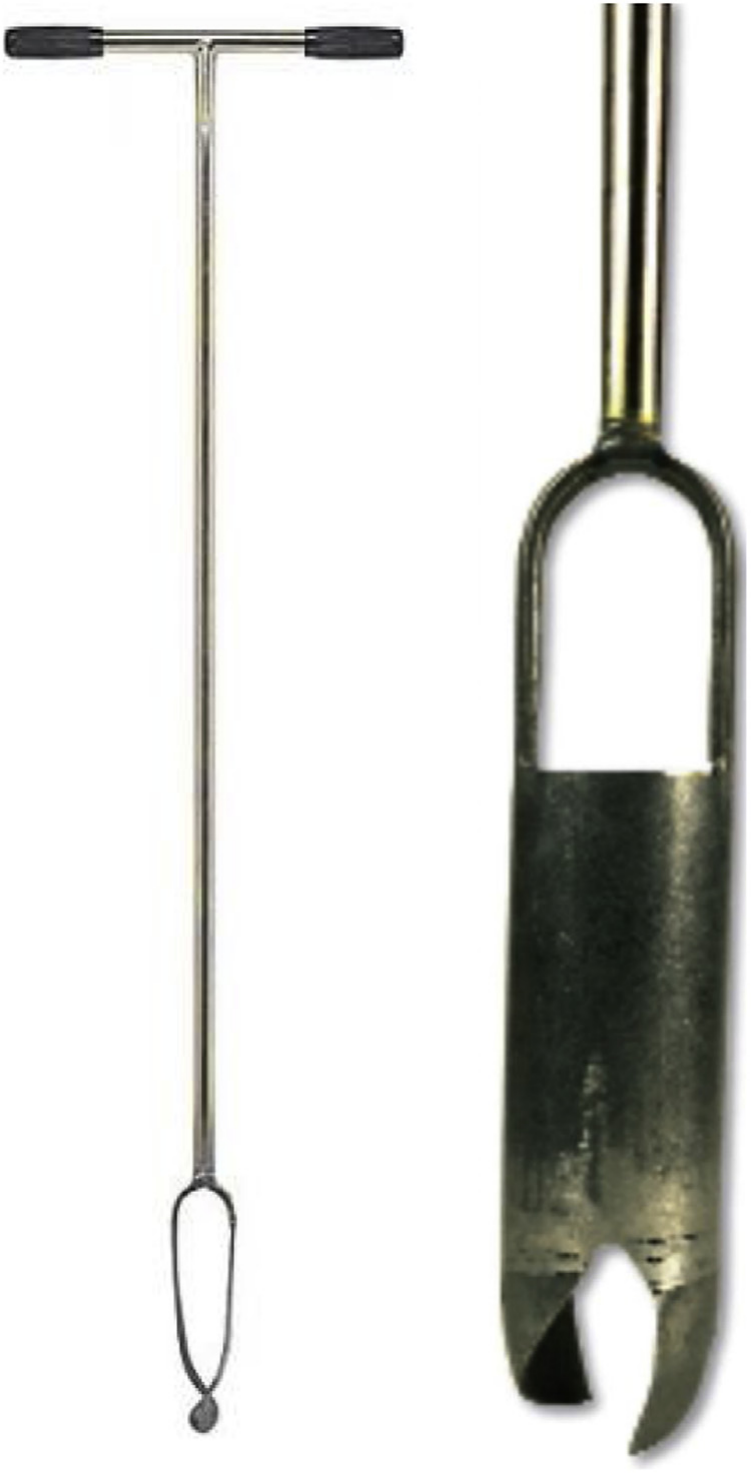
Soil sampling tools. Left – Dutch auger and Right – Bucket auger.
• Pre-labelled heavy duty polyethylene bags (see information for bulk density described above)
• Wire brush
• Soil knife
• Perforated drum grinder with 2 mm perforations
• Tape measure

**Equipment needed**:

• Bucket auger (also known as barrel and core auger) for dry, coarse textured soil and Dutch auger for wet, finer textured soil (Fig. 5)

**Procedure:**

*Field:*

• Use the bulk density samples for chemistry of soils to 30 cm depth.
• For the deeper samples, drill the auger tip into the bulk density sample hole by turning the handle in a clockwise rotation to the desired depth (30–60 cm and 60–100 cm). The soil is forced into and retained in the auger.
  ○ Be prepared to discard cores that are unrepresentative (e.g., excessively compacted during sampling, evidence of rodent activities and obstructed by rocks).
  ○ Remove any surface materials that have fallen into the hole before starting the collection for the next depth.
• Empty the soil into the labeled bag, avoid any loss of soil.
  • Note that you only need to keep a representative subsample of each depth range – otherwise you will end up with excessive amounts of soil.
• Carefully place the auger in the same hole and repeat the process until the desired depth is reached (use tape measure to measure depth).
• Store the sample in polyethylene bag in a large durable plastic bag for transport.
  • Store samples in a cooler for transport to the laboratory.

*Laboratory:*

• Store samples in a cool room (0–5 °C) until they can be processed.
• In the laboratory, remove soil from the polyethylene bags and air dry in lined trays at 37.5 °C. Avoid sample losses during processing and contamination by dust, plant material, and other contaminants.
• Once the samples are air dry, crush and grind the samples to pass a 2 mm sieve and screen out any rocks that are > 2 mm in diameter.
• Thoroughly mix the 5 core samples after they have been coarsely ground to < 2 mm and then subsample the soil for SOC, TN, EC and pH analysis.

Soil sample handling and storage requirements are provided in Table 1.

**Table 1:** Soil sample handling and storage requirements for the selected soil parameters.

Parameter	Sample grinding	Moisture	Storage before analysis	Archival Storage Conditions
Bulk Density	Avoided	Generally reported on an oven-dried basis	Indefinite if refrigerated, may change upon freezing	Indefinite if refrigerated, may change upon freezing
EC, pH, Organic Carbon and TN	Aggressive grinding acceptable to 2 mm	Generally reported on an oven-dried basis	Short term refrigerated, indefinite if dried	Indefinite if dried

## Vegetation assessment protocols

### Shrubs and 2-dimensional cover

This protocol is designed to measure vascular plant vegetation at the level of vegetation groups (e.g., grasses, forbs), except for shrubs which are measured at the species level.

**Field Equipment Needed:**

• ABMI Ecological Site Classification Chart (see Appendix A)
• Plant Field Guide (one that is relevant to the area which you are studying)
• Plant press
• Datasheets #6 and #7

**Procedure:**

**Fig. 6:**
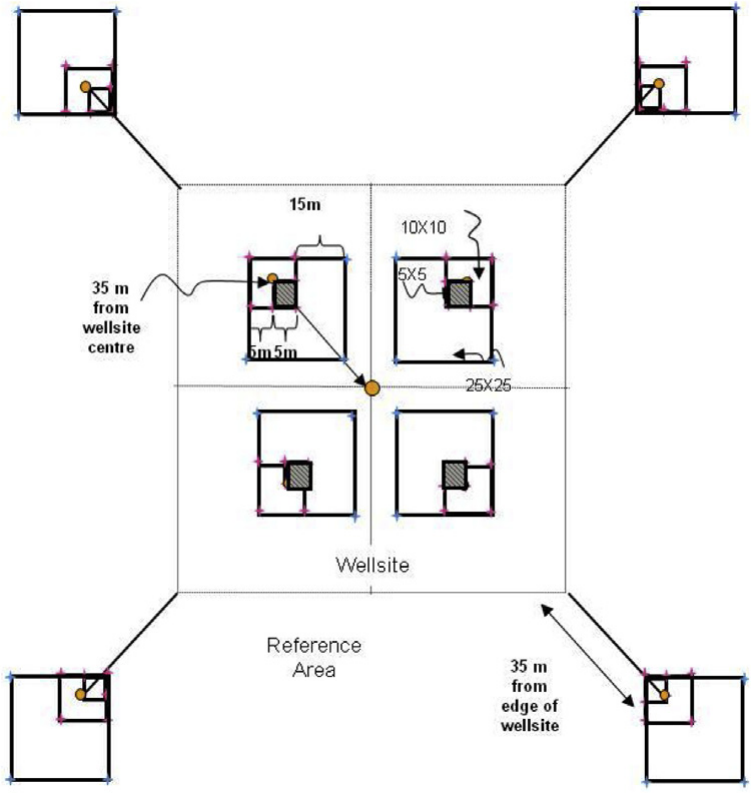
More detailed scale of 5 × 5 m, 10 × 10 m, and 25 × 25 m plot sampling. Shrub and 2-D cover are measured in the 5 × 5 m plots (shaded in grey) identified in the figure.
• Determine the ecological site type of each 5 × 5 m plot, using the ecosite classification chart (see Appendix A and Table 6 for full instructions).
• Describe slope position for each of the 5 × 5 m plots where vegetation cover is estimated as:
  ○ S = Slope – include a modifier (S1 for slopes 2-5°, as S2 for slopes 6-10°, as S3 for slopes 11-30°, and S4 for slopes > 30°).
  ○ C = Crest – situated in a relatively level area on the top of a hill.
  ○ T = Toe – situated at the bottom of a hill where the ground surface transitions from a slope to level.
  ○ L = Level – situated on in an area with < 2° slope.
  ○ D = Depression – situated in an area that accumulates water after rains.
• For all plots describe slope direction (looking down hill) in degrees.
• For the shrub layer estimate 2-dimensional cover (0, < 1, and 5% increments) of shrubs and seedlings/saplings^9^ .
  • Shrubs are defined as non-tree woody vascular plants that have woody stems.
  • Seedlings/saplings are defined as trees < 1.3 m in height and are included with shrubs in the estimates.
  • Shrub/seedling/sapling cover is estimated for three height categories (0 to 0.5, 0.5–2 m, and 2–5 m high). Note: Each of these estimates cannot be greater than 100%.
  • The estimate for height class 0.5 to 2 m is recorded as if a photo was taken 2 m above the ground and foliage from all shrubs/seedlings/saplings < 0.5 m was excluded.
  • The estimate for height class 2 to 5 m is recorded as if a photo was taken 5.0 m above the ground and foliage from all shrubs/seedlings/saplings < 2 m was excluded.
• For the ground layer (< 0.5 m), estimate 2-dimensional cover (0, < 1, and 5% increments) as the percentage of the 5 × 5 m plot covered by shrubs/trees, grasses (including sedges/rushes), all “other” vascular plants combined (herbs/forbs), mosses (includes all bryophytes), lichens, fungi, litter (dead vegetation material plus downed woody debris (DWD) < 2 cm in diameter), wood (live and dead trees > 1.3 m tall, plus DWD > 2 cm diameter), water, bare ground, rock, and animal matter.
  • These estimates are recorded as if a photo was taken 0.5 m above the ground.
  • Values of all these independent categories summed can exceed 100% for the strata < 0.5 m because of overlap among categories.
  • Record on Datasheet #6 (Appendix B).
• Record percent cover for each individual shrub/tree species rooted within the plot (regardless of size), including which strata (Table 2) it is located in.
  • Percent cover is determined by ocular estimation (this requires practice before the start of the data collection to ensure the estimates are precise).
  • Record on Datasheet #7 (Appendix B).

• 2-dimensional cover of the ground layer (Datasheet #6 Appendix B) and shrub layer (Datasheet #7 Appendix B) is measured at each 5 × 5 m plot (n = 9 5 × 5 m plots total, Fig. 6 – shaded boxes highlight the 5 × 5 m plots).

**Table 2:** Description of vegetation strata as described in the Ecological Land Site Description Manual (Alberta Sustainable Resource Development, 2003)^a^.

Code	Strata	Definition
T1	Tree (main canopy)	Trees that make up the upper part of the height distribution population and form the general layer of the canopy or foliage
T2	Tree (understory)	Trees and/or shrubs whose crowns extend into the bottom of the general level of the canopy or are located below the main canopy. Trees and/or shrubs must exceed 5 m height
S1	Shrub (tall)	All woody plants between 2–5 m tall (includes seedlings/saplings)
S2	Shrub (medium)	Shrubs and seedlings/saplings between 0.5–2 m tall
S3	Shrub (low)	All woody plants up to 0.5 m tall
H	Herbs (forbs)	Record all forb species regardless of height
G	Grass/graminoid	Record graminoids (grasses, sedges, rushes)
M	Moss	Record all bryophytes
L	Lichen	Lichen species growing on dominant substrate (usually mineral or organic soil) included
E	Epiphytes	Lichens or mosses growing on other plants, usually trees or shrubs
F	Fungi	Fungi (excluding lichen) growing on dominant substrate – mushrooms

a The S1 to S3 strata categories could be further subdivided into shrub and seedling/sapling sub-categories if finer detail is desired.

### Plant and lichen cover by species (0.25 m^2^ plots)

This protocol is designed to monitor relative abundance of vascular, non-vascular, and lichen species by height strata.

**Field Equipment Needed:**

• Plot frame (0.5 m × 0.5 m)
• Plant press
• Vascular plant field guide (one that is relevant to the area which you are studying)
• Datasheets #8A & #8B

**Procedure:**

**Fig. 7:**
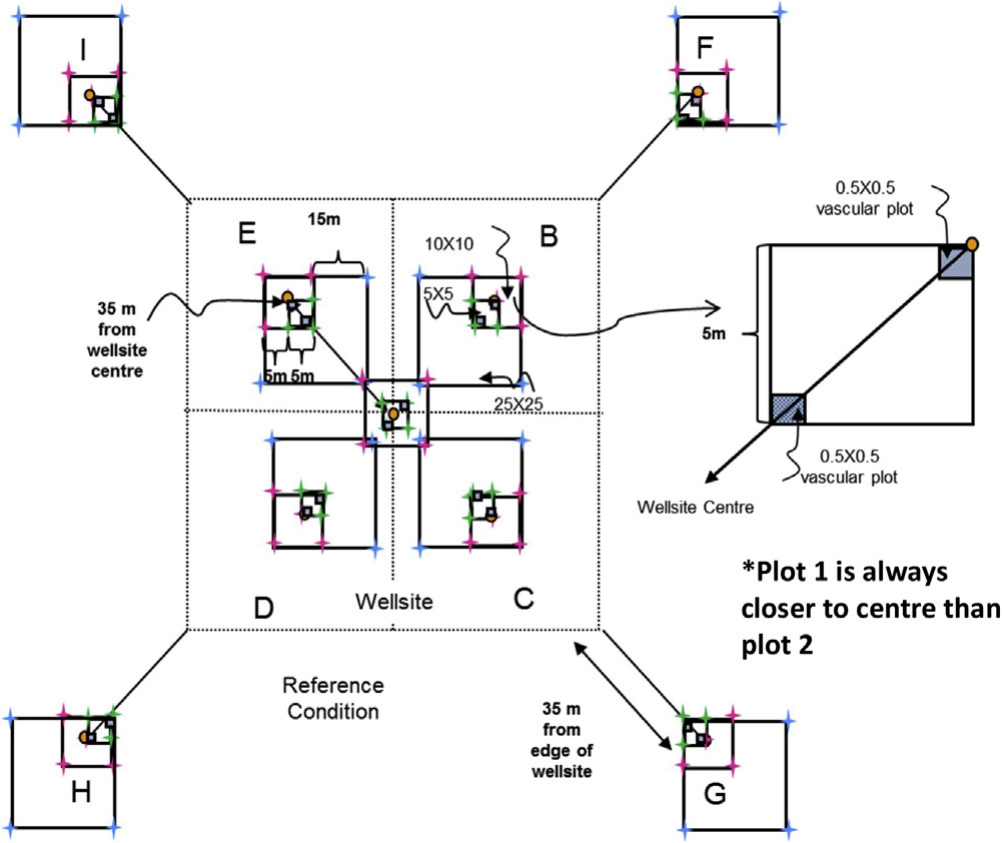
Locations of the 0.5 × 0.5 m quadrats where vegetation is sampled at the species × height strata level. Note that the wellsite centre 0.5 × 0.5 m plots are always in the B and D quadrants.

Ten plant and lichen cover quadrats (0.5 × 0.5 m = 0.25 m^2^) are established in the wellsite, and eight plant and lichen cover quadrats are established in the reference area (Fig. 7). For both the wellsite and reference area two 0.5 × 0.5 m cover quadrats are located in each of the 5 × 5 m plots at the two diagonal corners of the plot that intersect the sub-ordinal transects (see Fig. 7).

• Percent cover of individual vascular, non-vascular, and lichen species by strata are recorded within each 0.5 × 0.5 m quadrat. The strata are described in Table 2.
  ○ Use the same order of species list on the reference datasheet at a site as you did for the wellsite – then add additional species not found on the wellsite below this list (this will be helpful when data are being entered so the species data match up).
  ○ Record on Datasheets #8A and #8B.
• Estimate percent cover (0, <1, and 5% increments) by strata (see Table 2) for each species in each of the 0.5 × 0.5 m quadrats (Fig. 3).
  • Plants must be rooted within the quadrat to be included in the estimates.
  • Due to overlapping of leaves at different heights, percent cover for each species, and all species combined can be greater than 100%.
• In addition, estimate percent cover for rock, bare mineral soil, litter, and water in the quadrat.
• Collect voucher specimens of unknown or uncertain specimens from outside the 5 × 5 m plot if possible. Take the voucher specimens to camp for identification – be sure to properly label them so you can match them up with your datasheet.
  • When collecting voucher specimens, record the reclamation site number and a unique reference code (UIS-Site Number- Specimen Number) and collector’s name on the field data sheet and on the sheet in the plant press (e.g., the fifth unidentified specimen from site 1 in the DMG region would be: UIS-DMG-1-05).
• Place specimens that cannot be identified at camp that evening in a plant press for temporary storage. Ensure that the information (site number, plot (if applicable), reference code, date, collector’s name) on the data sheet matches the information included with the specimen in the plant press.
• Any plants that are identified at camp are discarded, the UIS line on the data sheet crossed out, the species code indicated beside the row, and a new row added for that species with all of the appropriate information added to the species record.
• At the end of the season, take the press to the laboratory. These unknown specimens will be identified by experts (see Processing of Specimens and Samples in Section 8.8).

### Vascular plant searches

This protocol is designed to detect as many species of vascular plants as possible during a time constrained search within the wellsite area along with the reference site. To standardize sampling effort a single person completes all of the vascular plant surveys at a site, in the time specified. It is recommended that this be done after the 0.5 × 0.5 m quadrats are measured so that the observer is already familiar with and identified some of the species.

**Field Equipment Needed:**

• Datasheet #9 (Appendix B)
• Plant field guide (only for use before or after timed searches)

**Procedure:**

*Wellsite Survey*

**Fig. 8:**
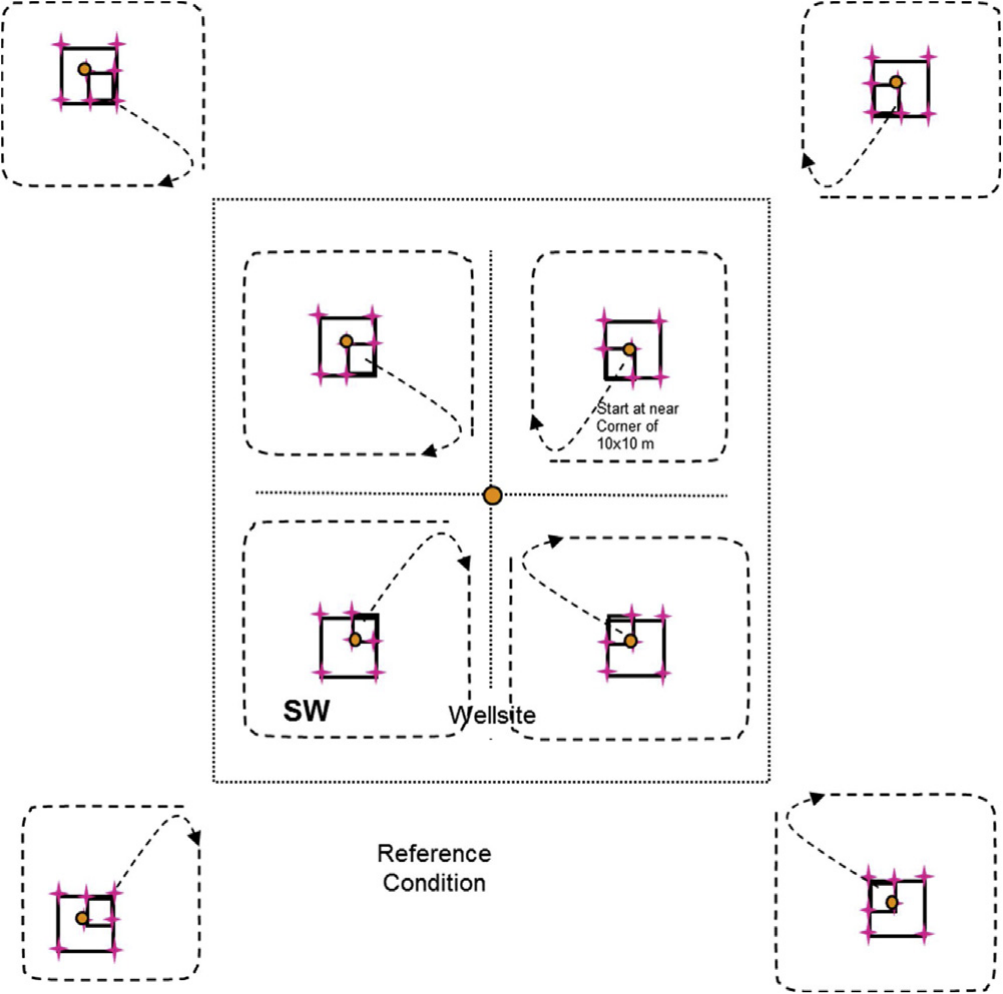
Layout of survey to identify vascular plant richness within the wellsite and reference areas.
• When a vascular plant species is detected in a quadrant, place a tick mark for that species in that quadrant on Datasheet #9 (Appendix B).
• Always start the surveys in the NE quadrant (B) and progress clockwise to the next quadrant (SE-C, SW-D and NW-E).

• The crew member surveying vascular plants spends an initial 10 min populating a species list with the names of vascular plants seen at the wellsite. This initial listing of plant names is conducted so that the subsequent timed searches of the 50 × 50 m quadrants are spent mainly looking for species, with less time recording plant names/codes.
  ○ During the initial 10 min when species are being recorded, locate the most diverse habitat types within the 1 ha site and spend time in these habitats recording species names.
  ○ Record on Datasheet #9 (Appendix B).
• The crew member then spends 20 min in each of the four quadrants (B to E; a total of 80 min) finding as many species of vascular plants as possible while walking a predetermined path (Fig. 8).
  • To maintain consistency among observers, start at the 10 × 10 m plot centre, and then begin heading toward site centre, to within 5 to 10 m. Then head in a clockwise direction around the quadrant staying approximately 5 to 10 m from the quadrant edge.
  • Stop every 4 or 5 steps to examine the plants in the immediate area (see Fig. 8).
  • Ensure that all habitat types in the quadrant are searched for vascular plants.

*Reference Area Survey*

• The crew member surveying vascular plants uses the plant species list developed in the wellsite survey and adds easily identifiable species found in the reference areas.
• The crew member then spends 20 min in each of the four ‘quadrants’ (50 × 50 m = 2500 m^2^ – dimensions will vary depending on shape of reference area polygon) (F to I; a total of 80 min) finding as many species of vascular plants as possible while walking a predetermined path (Fig. 8).
  ○ Record on Datasheet #9 (Appendix B).
• To maintain consistency among observers, start at the 10 × 10 m plot stake, and then begin heading toward the edge of the wellsite, to within 5 to 10 m. Then head in a clockwise direction around the ‘quadrant’ staying approximately 5 to 10 m from the quadrant edge.
  • Stop every 4 or 5 steps to examine the plants in the immediate area (Fig. 8).
  • Ensure that all habitat types in the quadrant are searched for vascular plants.
• When a vascular plant species is detected in a reference ‘quadrant’, place a tick mark for that species in that quadrant on Datasheet #7 (Appendix B).

*For Unidentified Species:*

• If the crew member is unable to identify a species quickly during the 10-minute search they will collect the specimen from a population of greater than 5 individuals, outside the plot if possible.
  ○ These samples are assigned a unique specimen number and carried with the crew member so as to avoid multiple collections in each quadrant if possible.
  ○ Unidentified specimens are named UIS-Site Number-Wellsite/Reference-Specimen Number e.g., UIS-3-W-1.
• Field guides should not be used during the 20-minute search time.
• Collect voucher specimens of unknown or uncertain vascular plant species. After the 20-minute search in a quadrant is complete, attempt to quickly identify the species you have collected using field guides.
• Place labeled unknown specimens in a plant press and take them to camp for identification during the evening.
  • The label on the specimen tag and in the plant press log will be written as UIS-Site Number-Wellsite/Reference-Specimen Number (e.g., the fifth unidentified specimen from site 3 in the wellsites would be: UIS-3-W-5).
  • Ensure that specimen numbers are not repeated for the site, especially for specimens from the low vegetation and shrub cover plots.
• For any vascular plant categorized as S1 or S2 by Alberta Natural Heritage Information Centre (ANHIC), collect a specimen so its identity can be confirmed by experts.
  • Collect the specimen from a population of greater than 5 individuals, outside the plot if possible.
• Place specimens that cannot be identified in the evening, or ANHIC S1 or S2 plants, in the camp press.
• Any plants that are identified at camp are discarded and the UIS number will be removed and replaced with the correct species code. Do not forget this step.
• Any species found after the vascular plant search is complete are to be recorded under incidental species.
• At the end of the field season (or sooner if the plant press is full), deliver plant presses to the lab. These unknown specimens will be identified by experts (see Processing of Specimens and Samples in Section 8.8).

### Trees, snags and stumps

This protocol is designed to measure tree (> 1.3 m in height), snag, and stump densities and sizes and as such will only be relevant when there are trees present.

**Field Equipment Needed:**

• 5 m DBH tape
• 10 m carpenters tape
• 100 m measuring tape
• Vertex hypsometer w/transponder – see Appendix A for instructions on calibration and use (or clinometer if hypsometer is not available)
• Tree paint
• Datasheet #10 (Appendix B)

**Procedure:**

**Fig. 9:**
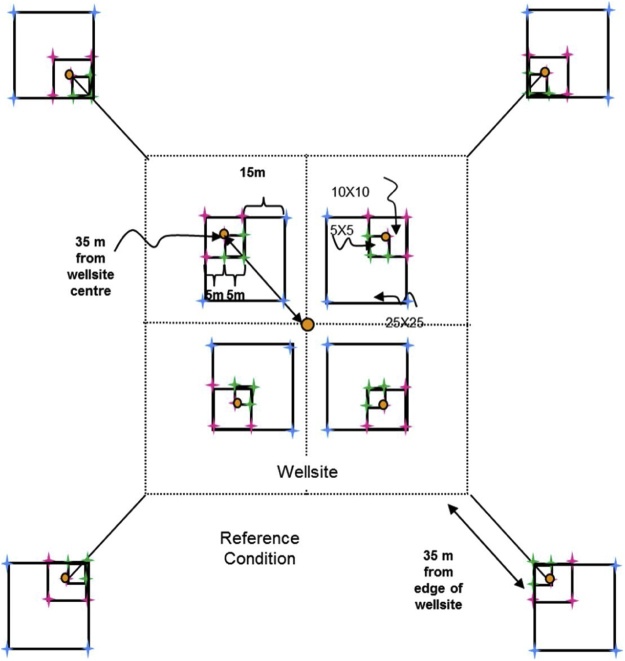
Detailed layout of 5 × 5 m, 10 × 10 m, and 25 × 25 m tree, snag, and stump sampling.
• The second plot is 10 × 10 m (Measure ALL trees snags and stumps ≥ 7 cm diameter), and encompasses the entire 10 × 10 m plot (Fig. 9).
  ○ Do not remeasure the trees from the 5 × 5 m plot above – instead complete measurements of the 3 remaining 5 × 5 m plots within the 10 × 10 m plot – but for only trees > 7 cm diameter.
• The third plot is 25 × 25 m (Measure ALL trees snags and stumps ≥ 25 cm diameter) and encompasses both the first and second plots (Fig. 9, Fig. 10).

Data are collected in three nested plots for three different size categories (record on Datasheet #10 Appendix B):

• The smallest plot is 5 × 5 m (Measure ALL trees, snags and stumps) and is anchored at the centre of the 10 × 10 m plot located at each quadrant centre – with the 5 × 5 m plot in the quadrant of the 10 × 10 m plot that is closest to the wellsite centre (Fig. 9).

**Fig. 10:**
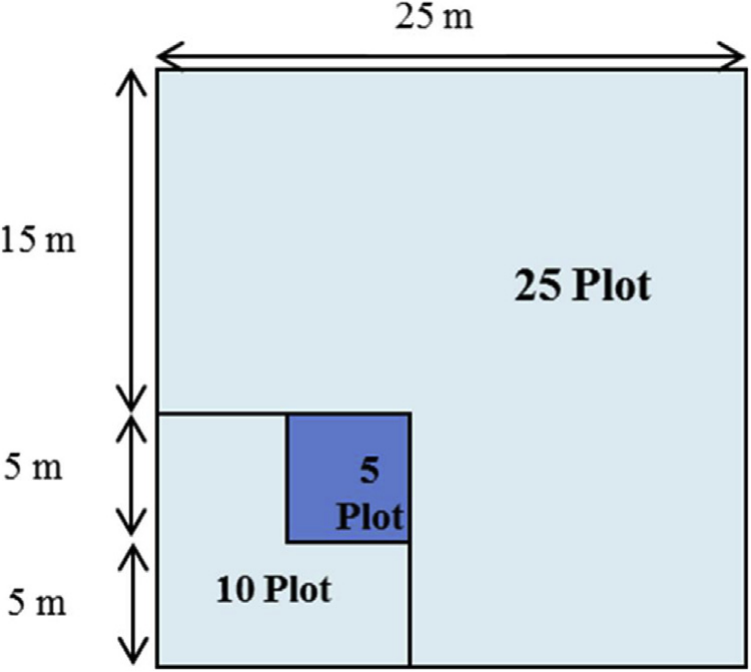
Tree, snag, and stump plot layout of wellsites and adjacent reference areas delineating the 3 different plot types. The 5 × 5 m square plot is located in the quadrant of the 10 × 10 m square plot that is closest to the wellsite plot centre (see Fig. 9).
• For accurate and consistent data collection treat the 5 × 5 m plot as a square, and the 10 × 10 m and 25 × 25 m plots as L-shaped (Fig. 9, Fig. 10).
  • Collect the information in the 5 × 5 m and then sequentially move to the 10 × 10 m and then 25 × 25 m plots.

*For the purposes of this protocol:*

• Trees are defined as any tree species with the exception of Alnus or Salix that is ≥1.3 m in height.
  ○ Trees < 1.3 m in height (i.e., saplings) are NOT measured.
• Snags are defined as DEAD trees ≥ 1.3 m in length, leaning ≤ 45°from vertical. Snags can be intact or broken below the canopy.
• Stumps are defined as DEAD trees broken below the canopy with a top height of < 1.3 m. Height of all stumps is measured to the nearest 0.1 m.
• Diameter is measured to the nearest 0.1 cm using a DBH tape.
• Top height and base height of trees and snags are measured to the nearest 0.1 m unless this value is estimated, in which case height is estimated to the nearest 0.5 m.
  • Tree height is measured using a vertex hypsometer, or in the case of small trees, can be measured using a carpenter’s tape.
  • If a hypsometer is unavailable then a clinometer and tape can be used to estimate height.
• Measure trees and snags on the boundary of the plot only if greater than half of the bole is within the plot.
  • Note that flagging tape may not be an accurate determination of plot boundary; if in doubt crews should lay out the measuring tape to confirm plot boundaries.
• Mark each sampled tree and snag at breast height (i.e. point of measurement) with a small dot of tree paint to avoid re-sampling.
• Further details on the sampling within each of the three plots are described below (see Fig. 9, Fig. 10 above for clarification of location of each plot).

*5 × 5 m Plot (5 – square)*

**Fig. 11:**
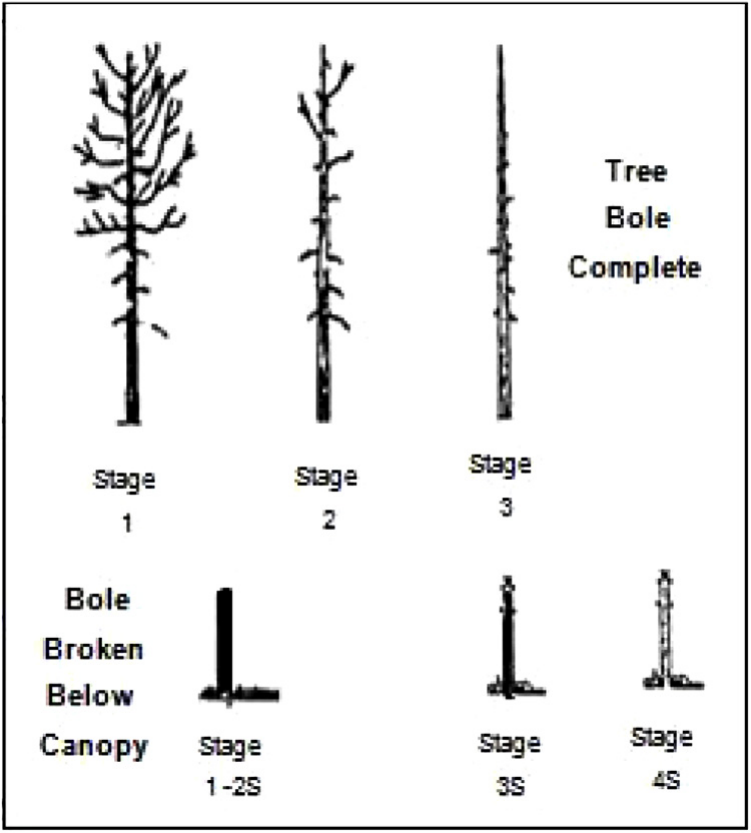
Visual representation of decay stages for snags and stumps.
• For snags > 7 cm DBH, record the same categories as live trees, but include the appropriate decay stage, and exclude Crown Class.
• For stumps, record Plot, Species, Condition, Decay Stage, and Top Height.

• Measure ALL trees, snags, and stumps.
• For trees < 7 cm DBH, record Plot Type, Species, Condition, and DBH.
• For trees ≥ 7 cm DBH, record Plot Type, Species, Condition, DBH, for all trees and Tree Height for the 3 tallest trees.
• For snags < 7 cm DBH, record the same categories as live trees, but include the appropriate decay stage (described in Table 3 and Fig. 11).

**Table 3:** Description of decay stages.

Decay Stage	Description
Trees not snapped	
1	Recently killed, all twigs/ branches present, wood hard, bark (normally) intact
2	Twigs and small branches missing (major branches remain), wood hard
3	No branches, bole mostly intact, wood starting to soften.
Tree snapped along bole: twigs and branches absent	
1-2S	Recently killed, wood hard, bark (normally) intact
3S	Wood starting to soften
4S	Wood soft throughout the snag

Refer to Fig. 11 for visual representation of decay stages.

*10 × 10 m Plot (10 – L-shaped)*

• Measure ALL trees snags and stumps ≥ 7 cm DBH (trees/tall snags) or diameter (short snags and stumps).
• For all trees, record Plot Type, Species, Condition, DBH, and for the 3 tallest trees measure Tree Height.
• For all snags, record the above categories, including the appropriate decay stage.
• For stumps, record Plot Type, Species, Condition, Decay Stage, Diameter, and Top Height.

*25 × 25 m Plot (25 – L-shaped)*

• Measure ALL trees, snags, and stumps ≥ 25 cm DBH (trees/tall snags) or diameter (short snags and stumps).
• For all trees and snags, record Plot Type, Species, Condition, Decay Stage, DBH, and for the 3 tallest trees measure Tree Height.
• For stumps, record Plot Type, Species, Condition, Decay Stage, Diameter, and Top Height.

### Tree cores

This protocol is designed to measure age, and growth rate of trees (when trees are present).

**Field Equipment Needed:**

• 16 inch (5.5 mm) increment borer
• Straws
• Stapler – to seal the end of straws – tape should not be used as otherwise cores will be moldy
• Ziploc bags
• Labels
• Vertex hypsometer (see Appendix 6 for instructions on calibration and use)
• Folding saw
• DBH tape
• Datasheet #11 (Appendix B)

**Procedure:**

The following applies to both the wellsite and reference area.

• Tree cores should be obtained from 4 of the same live trees measured for height.
• Tree species are selected based on their relative abundance, height and age.
• One of the selected trees should be that with the largest DBH.
• Record DBH, tree height, and significant tree damage for the selected tree with the largest DBH.
• Use a vertex hypsometer to determine tree height for each of the cored trees. Record height to the nearest 0.1 m.
• Use a DBH tape to record DBH to the nearest 0.1 cm.
• Significant tree damage is defined as any damage or condition that could affect the normal height or growth rate of the tree: broken tops, dead tops, forks, crooks, and/or abnormal scarring or other damage (e.g. mistletoe). Record significant damage as:
  ○ BT – Broken Top,
  ○ DT – Dead Top,
  ○ FC – Fork/Crook,
  ○ S – Scarring, and/or
  ○ O – Other (indicate damage from diseases, insects, wild and/or domestic animals, abiotic natural factors, and anthropogenic factors).
• Use the increment borer to obtain the cores. Bore the tree at 1.3 m (be accurate when determining the height of the core) and facing site centre, if possible.
  • If the tree is not round, obtain the core from the narrow width.
• If core is rotten or breaks into more than 2 pieces while being extracted, make one more attempt to collect a core sample. If this fails, collect a core from another similar tree.
  • Where a core cannot be obtained due to rot, record the DBH, height, and significant tree damage of the tree in question and indicate that a core was not collected and why (e.g. Other-with comments, rot).
• Preserve the core in a straw.
  • Staple the straw ends (do not tape) to ensure the core can dry.
  • Puncture straw in many places to allow air flow and stop mold/rot.
• Label the core with the following information: site, quadrant, tree species, sample type (largest, leading, second), initials, and date.
• If all trees in the 50 × 50 m quadrant of either the leading or secondary species are < 10 cm DBH, destructively sample a representative tree from outside of the quadrant by taking a cookie at a height of 1.3 m.
  • This makes it possible to core a leading species and take a cookie from a secondary species, or vice versa.
• If all trees in 50 × 50 m quadrant (excluding veterans) are < 10 cm DBH, only destructively sample the leading tree species from outside of the 1 ha area (i.e., total of 4 trees per site).
  • Place the cookie in a paper bag. Cookies left in plastic bags will rot.
  • Place a label in the bag or write on the outside with the same information as above.
  • Note the secondary species, but do not take a second cookie from outside the quadrant.
  • Place all cores in a protective case to transport from site to camp; be especially careful not to break the cores.
• When back at camp, dry the cores in a warm and dry environment to avoid rot.
• At the end of the shift, pack samples in a cardboard box and take them to the laboratory for processing (see Processing of Specimens and Samples in section 8.8).

### Canopy cover

This protocol is designed to measure canopy cover within the site (and should only be recorded when there is vegetation that is taller than breast height (1.3 m).

**Field Equipment Needed:**

• Spherical (concave) densiometer
• Datasheet #12 (on same sheet as Datasheet #11 Appendix B)

**Procedure:**

**Fig. 12:**
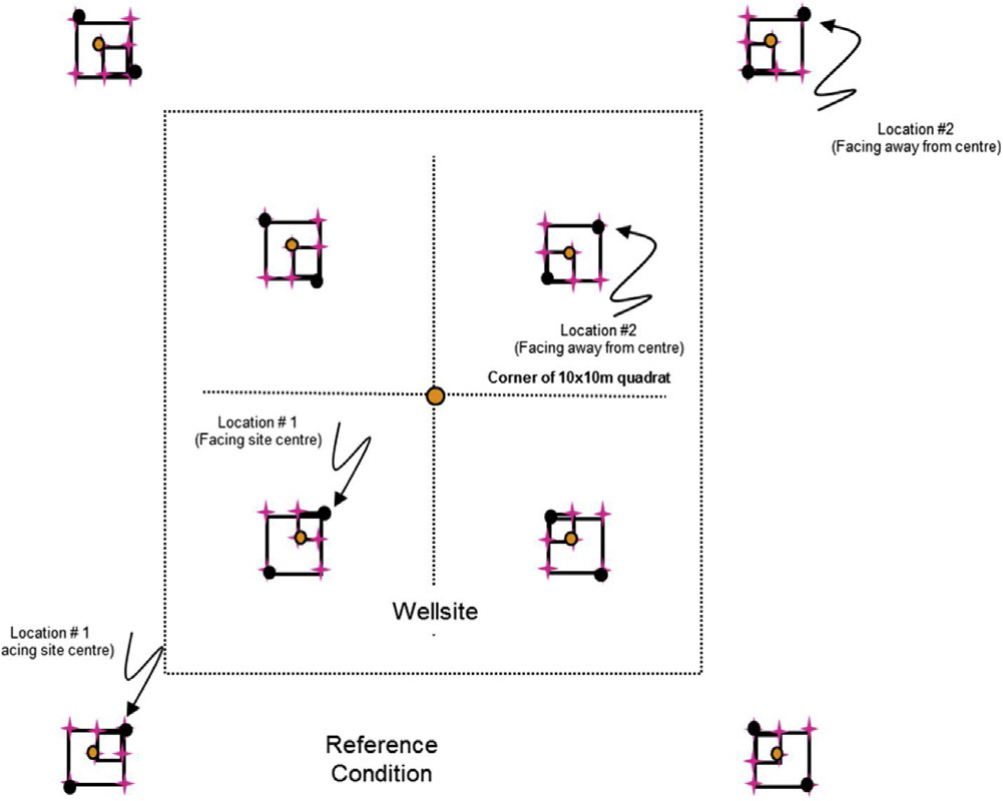
Location of points where canopy cover is measured. Black dots show measurement points.

**Fig. 13:**
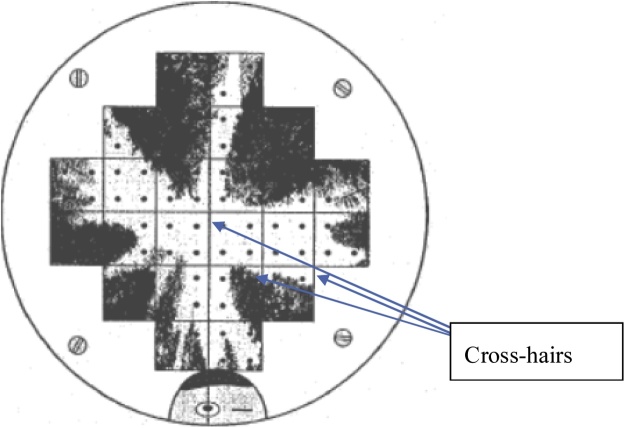
View of densiometer mirror. Placement of observer’s head and the 37 cross-hairs are shown. In this example there are 12 out of 37 cross-hairs that are open. Note: ignore the dots in the diagram as they are not relevant for the way that we are recording cover data. Diagram Diagram taken from Shuett-Hames et al.

• Take densiometer readings at the two diagonal corners of wellsite quadrants B and E and reference quadrants F and I 10 × 10 m plot along each sub-ordinal transect (a total of 16 readings per site – see Fig. 12).
  ○ Hold the densiometer in the palm of your hand at elbow height (i.e., with your arm bent at right angles) and ensure that it is level (bubble is in the middle of the circle).
  ○ Stand facing site centre at the corner that is closer to wellsite centre, and stand with your back to site centre at the corner that is furthest from plot centre.
  ○ Count the number of cross-hairs (out of a total maximum of 37) that are in canopy openings (i.e. NOT covered) and record the number of open cross-hairs on the Datasheet #11 (Appendix B; see Fig. 13).

## Woody debris sampling

### Downed woody debris (DWD) sampling

**Field Equipment Needed:**

• cm DBH Calipers
• Go-No-Go Tool
• Mora Knife
• 50-m Measuring Tape
• Datasheet #13

**Procedure:**

**Fig. 14:**
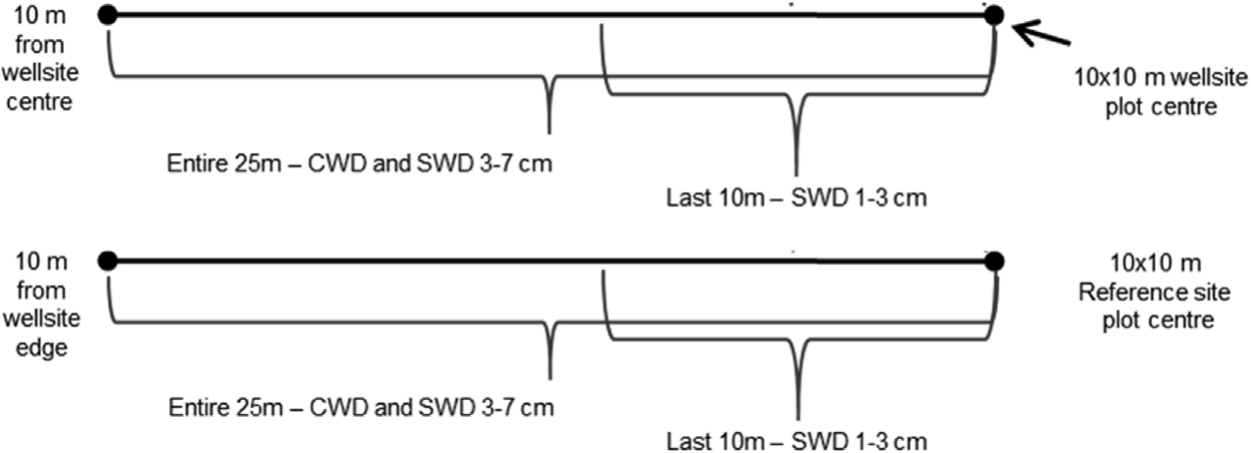
Delineation of sampling of small (SWD), and coarse woody debris (CWD) along 25 m transects for a) wellsite transects and b) for reference site transects.

**Fig. 15:**
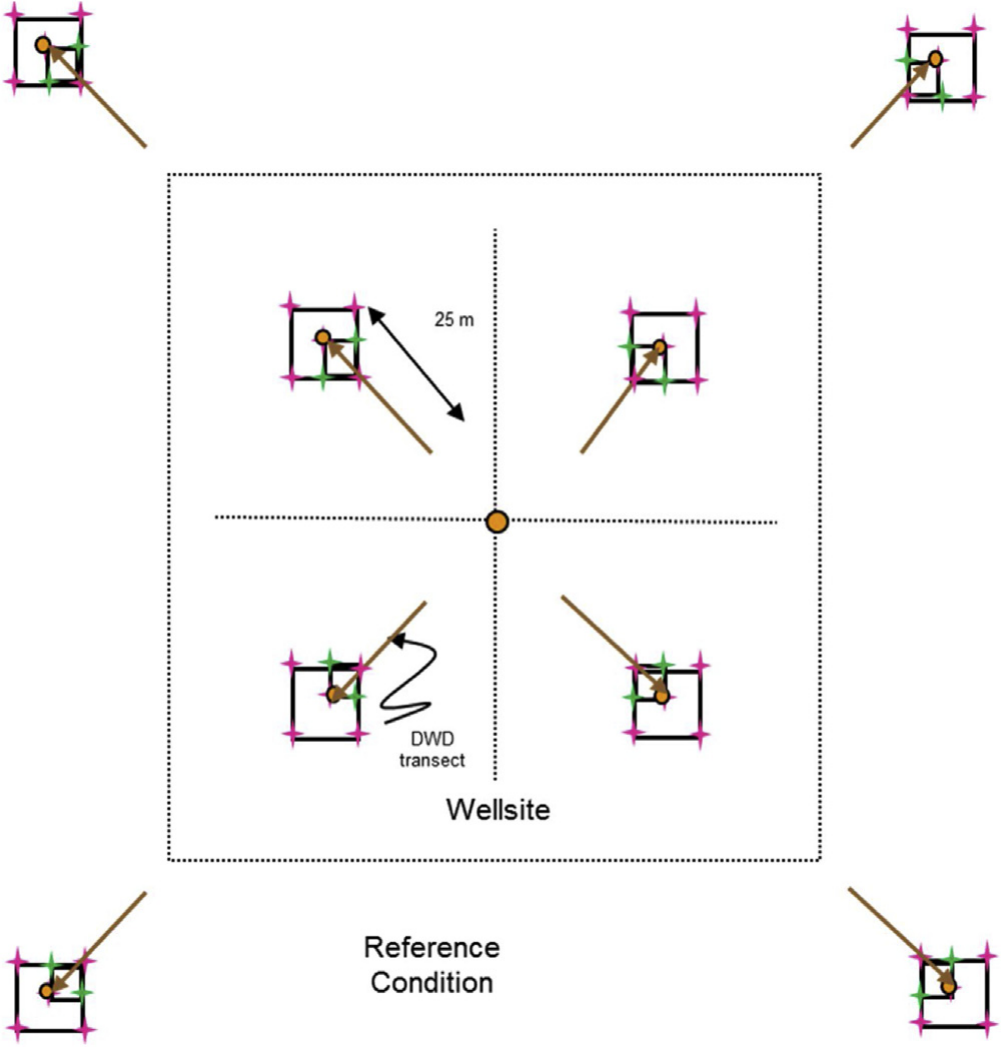
Transect locations where DWD is sampled in the wellsite and reference sites – transects are highlighted in brown.
• Dead wood must be on the forest floor or leaning > 45° from vertical to be recorded as DWD, otherwise it is recorded as a snag (see vegetation section of protocols).
• DWD is divided into two categories (the “go-no-go” tool is used to determine which category each piece belongs to):
  ○ Coarse Woody Debris (CWD; ≥ 7 cm)
  ○ Small Woody Debris (SWD; 1–7 cm - (split into 3 size classes: 1.0–3.0, 3.1–5.0 and 5.1–7.0 cm))
  ○ Note that Fine Woody Debris (FWD; ≤ 1 cm) is not recorded as that information is captured in the litter estimates that are recorded in the 0.5 × 0.5 m vegetation plots.
• Along the entire 25 m of each DWD transect measure each piece of CWD > 7.0 cm diameter (in 0.5 cm increments) where it intersects the transect:
  • Record decay stage on Datasheet #13 (see Fig. 16)

• DWD is measured on the four sub-ordinal transects for wellsite quadrants B–E and reference quadrants F–I.
• Transects start 10 m from the wellsite centre in each quadrant and extend 25 m to the pigtail located at the middle of the 10 × 10 m square in each quadrant for the wellsite transects. For the reference sites, the plots run from the pigtail located 10 m from the edge of the wellsite out 25 m to the pigtail located in the middle of the 10 × 10 m square (see Fig. 14, Fig. 15).

**Fig. 16:**
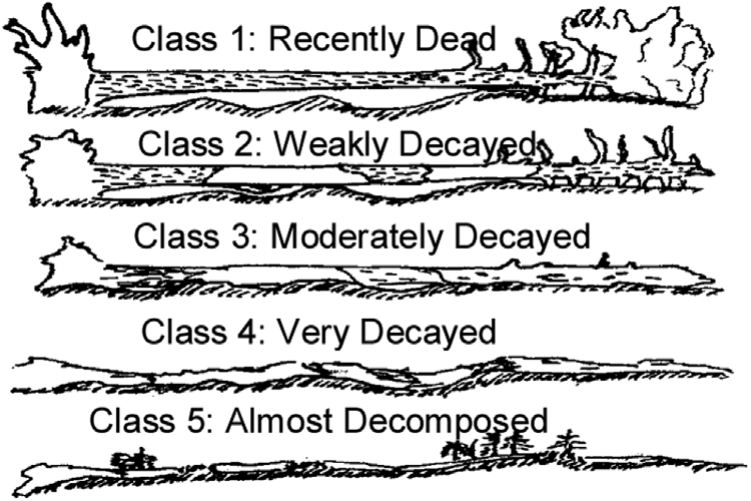
Visual representation of decay stages for coarse woody debris. Only Classes 1–4 are recorded.
  • Record diameter at point of intersection with the measuring tape. Measure diameter using DBH calipers in a plane perpendicular to the long-axis of the CWD.
• SWD is placed into 3 size classes (1.0–3.0, 3.1–5.0 and 5.1–7.0 cm).
  • SWD 3.1–5.0 cm and 5.1–7.0 cm are tallied along the entire 25 m transect. To be included as SWD, the piece must intersect the transect and be above the litter layer (i.e., < 50% buried).
  • Tally the number of SWD 1.0–3.0 cm along ONLY the last 10 m (i.e., from the centre of the wellsite 10 × 10 m plot to 10 m closer to the wellsite center) of each of the 8 DWD transects.

When classifying the state of decay (1–4), careful attention should be given to all criteria listed (Table 4).^10^

**Table 4:** Description of downed woody debris (DWD) decay classes.

DWD Class	Description
1 – Recently Dead	Bark (normally) attached to the wood; little or no fungal mycelium developed under patches of loose bark. (*100 to 95% of the initial dry density*)
2 – Weakly Decayed	Loose bark (intact or partly missing); well-developed fungal mycelium (normally) between bark and wood; rot extends <3 cm radially into the wood (as measured by pushing a knife into the wood). (*˜ 95 to 75% of the initial dry density*)
3 – Moderately Decayed	Rot extends >3 cm into the wood (as measured by pushing a knife into the wood) but core still hard; log may be sagging or broken but still supported from forest floor by stones, humps, etc. (*˜75 to 50% of the initial dry density*)
4 – Very Decayed	Rotten throughout (entire knife penetrates into wood); log shape conforms to forest floor; often elliptical in shape. (*˜50 to 25% of the initial dry density*)

In some cases it may be difficult to determine what to measure. The following provides some details to help clarify sampling procedures:

1 Classified as Downed Woody Debris:
  a Twigs, stems, branches, and chunks of wood > 10 cm long with or without bark.
  b DWD above the litter layer or soil; debris is considered no longer above when it is >50% buried beneath a layer of surface organic matter (forest floor) or mineral soil.
  c Odd shaped pieces of wood; estimate diameter at intersect as if it were round.
  d Fallen or suspended (not self-supporting) dead tree boles and branches, with or without roots attached, that intersect the plane of the transect line and are leaning > 45° from vertical. Stems and branches may be suspended on nearby live or dead trees, other coarse woody debris, stumps, or other terrain features.
  e Fallen trees/branches with green foliage that are no longer rooted in the ground (decay stage 1).
  f Large fallen branches and broken tree tops that are horizontal or leaning and not connected to the tree bole.
  g Recently cut logs.
  h Uprooted (not self-supporting) stumps.
  i Exposed dead roots of snags/logs that have fallen and are crossing the transect.
2 Things NOT classified as Downed Woody Debris:
  a Cones, bark flakes, needles, leaves and forbs.
  b Live or dead trees (still rooted) which are self-supporting and leaning < 45° from the vertical.
  c Dead branches still connected to standing trees.
  d Exposed roots of self-supporting trees.
  e Self-supporting stumps or their exposed roots.
  f A piece is no longer considered debris when the wood is decomposed to the point where it could be described as forest floor humus (no discernible shape of log left).
3 Accumulations of large DWD (e.g., logging debris or slash piles): If a pile of CWD is encountered along the transect, and it is too time consuming to measure each piece individually, then a portion of the accumulation is measured and the total estimated from that partial measurement.
  a For piles of DWD – If the pile is at an angle to the transect line, estimate perpendicular diameter at the point of intersect similar to what would be done for a log (note: do not measure pile width based on the intersect with the transect line). Estimate the horizontal perpendicular width of the pile, and the average vertical depth of the pile. Visually compress the pile to determine the actual cross sectional area of wood, not the space between the pieces. Based on length and width, estimate an approximate diameter of the accumulation as if it were round.
  b Identify and record the most common species in the accumulation and the most common decay stage.
  c Record an “A” under the accumulation column on the datasheet. Note that if no accumulations were present, VNA is recorded.
4 Partial Tally is used when many pieces of SWD cross the transect (e.g., wind throw and broken-off tree crowns containing many small branches). If a tree crown has fallen across the transect, a proportion of the branches/pieces are counted and the total number is estimated.
  a Measure the entire horizontal length of the debris field crossing the line (i.e., debris field is 5 m long).
  b Choose a representative sub-sample (not just the first portion of transect) and tally the number of SWD pieces (i.e., 25 pieces of SWD tallied within a 50 cm distance).
  c To obtain an accurate estimate of DWD, the length of transect chosen for measurement must have at least 20 pieces.
  d Estimate the number of pieces in the total debris field (i.e., in the above scenario multiply by 10; 250 pieces of SWD).
  e This is recorded in the partial tally section of the datasheet. Use VNA if you do not encounter a case which requires a partial tally.

### Managing personnel, data quality and integrity

This section provides background information related to the number of individuals needed to collect the data, the training field staff should receive prior to data collection, how datasheets should be completed in the field, including some metadata for the coding of data, ensuring data quality and completeness, procedures for storage and transfer of field-collected samples, and entry of data after it has been collected.

### Safety

All field crews and laboratory personnel are required to follow the safety procedures stipulated by their employers for the type of work being conducted and to comply with all provincial and national safety laws.

If at any time during the season you feel safety (of yourself or anyone else) is being compromised, tell a field coordinator immediately. Safety ALWAYS comes before the objectives of data collection.

### Personnel and sampling

These data collection protocols are optimally designed to be implemented by a field crew of two (2) personnel working together or, at times, semi-autonomously. At least one of the field crew members should be familiar with reclamation and reclamation practices and regulations.

### Crew training prior to data collection

All field staff are to receive proper and appropriate training so they can operate vehicles and equipment safely. In addition, staff are to receive extensive training (in the classroom and field) prior to the beginning of the field data collection. This protocol training includes learning what to do in the variety of field conditions that will be encountered, as well as conducting data collection at test sites. Crew members are first required to become familiar with the protocol documents, field manuals and general field procedures. Then they practice the data collection in the types of habitats where they will be sampling. Questions that arise during the training are discussed with the field supervisors. When possible, this training is provided by experts in the field.

At least one member of the crew should be trained in plant identification (especially for problematic species likely to be encountered such as grasses, mosses and lichens). Where this is not possible crews must be able to collect high-quality specimens for later identification.

Field crews are to review the protocols regularly to ensure that data collection remains accurate throughout the field season and nothing is being missed.

### Preparation prior to data collection

The plastic bags and labels for the soil sample collecting should be completed prior to going out in the field. Paper bags should be available for any vegetation specimens to be identified later.

A large waterproof bag that includes the datasheets and the sampling bags for each site should be organized and ready for collection of samples in the field. See additional sampling sections for additional information.

### Completing data sheets in the field

Crews are responsible for filling information into the data sheets while conducting field protocols (in the future data may be collected using tablets in place of field datasheets, but for now datasheets (rite in the rain) are used). Data should be reviewed by a supervisor before moving to the next site.

Data sheets must reflect exactly what was found/measured at the site. If options for the data field do not include an appropriate response, crews are instructed to record the most appropriate descriptors and make extensive notes on the data sheets. Technicians do not create new categories or descriptors. All fields on the data sheet must have information recorded – even if it is a “zero”, “not applicable”, “did not collect” (see below for description of each). If data could not be collected for a specific element, then this must be noted on the data sheet and the crew supervisor advised as soon as possible (note that supervisors must be notified by the end of the day at the latest).

**None or 0** – None or “0” is applied to any variable that was examined by field crews and found to be absent. “None” is used for text entries and “0” is used for numerical entries. Note: “0” can also be used as a code – for example, wind conditions can be recorded as “0”.

**Variable Not Applicable (VNA)** – VNA indicates that the cell cannot have data present.

**Did Not Collect (DNC)** – Use “DNC” to describe variables that should have been collected but were not due to crew oversight, equipment failure, safety concerns, environmental conditions, or time constraints. The use of DNC highlights that the cell ordinarily would have contained data.

### Checking field data and storing data sheets daily

Data sheets must be checked every evening for legibility and completeness. If data on a sheet cannot be corrected so they are legible, the data must be transcribed onto a new data sheet and both copies filed. Wet data sheets are allowed to dry, and then all data sheets are stored in a secured area if possible while in the field (e.g., in a folder in the trailer). Data sheets from one site cannot be taken to the field at another site. Crews must re-collect lost or missing data.

### Transferring field data sheets to a secure location

Data sheets are transferred in person to the crew supervisor when the supervisor visits, or at the end of a shift. The completeness (i.e., all data sheets present and all data fields filled in) of the data sheets is confirmed during the transfer. Missing fields or data sheets must be re-collected. Field supervisors take the data sheets to a secure office at the end of the shift, or sooner if possible. Data for each site are stored in a separate folder, with the folders organized by site number. Original data sheets are not allowed to leave the secure office.

### Processing of specimens and samples

Soil samples are transported by crew members to an accredited laboratory selected by the Program Lead.

Vegetation specimens are transported to the facility selected by the Program Lead for identification.

Chain of custody records must be maintained to track samples and specimens from field to laboratory.

### Data entry and verification

Data are entered into an electronic database. If data are entered at a different location than they are stored the data sheets are photocopied or scanned and data entry occurs from the copies. Data entry is verified by comparing the electronic information against the information on the original data sheet. Electronic verification routines are performed on the database to ensure that data are consistent with the allowable codes and among sites.

## Glossary of terms and acronyms

### Terms

#### Candidate site

A site within the universe of available certified sites that has a high rating based on the Appendix A, Table 5 criteria.

#### Clinometer

An instrument used for measuring the angle or elevation of slopes.

#### Hypsometer

An instrument for measuring height or elevation.

#### Monitoring site

An area of land subject to the Ecological Recovery Monitoring Program that includes:

1 Land that has been disturbed while conducting a specified land activity as defined in s. 1(t) of the Conservation and Reclamation Regulation [8]; and, has been certified by a government agency as being reclaimed pursuant to the requirements of the Environmental Protection and Enhancement Act [7] and the Conservation and Reclamation Regulation [8]; and,
2 The associated Reference Areas.

The area of land subject to the monitoring program may form all or part of the area occupied by the specified land activity and/or certified as reclaimed.

#### Opportunistic site

A site that has a lower rating based on Appendix A, Table 5 but is within a reasonable distance from a candidate site and could be added to the Program with minimal travel cost impacts.

#### Parameter

A specific characteristic such as plant height or bulk density that is evaluated as part of the Program.

#### Pilot program

A four-year research program (2012–2015) to determine the need for, and if required the design of, an integrated, scientifically robust and financially sustainable program for the long-term assessment of ecological recovery of certified reclaimed specified lands. Partners in the Program included Alberta Environment and Parks (AEP) (formerly the Alberta Environmental Monitoring, Evaluation and Reporting Agency [AEMERA]), the Alberta Biodiversity Monitoring Institute (ABMI), and InnoTech Alberta (formerly Alberta Innovates – Technology Futures [AITF]).

#### Plot

A sampling unit of varying size depending on the parameter of interest, usually a square e.g., 10 × 10 m for soils). Plots are located in the wellsite and in the reference areas.

#### Program

The Ecological Recovery Monitoring Program.

#### Quadrant

A sampled area that is 50 × 50 m (or may be slightly smaller if the wellsite was smaller than 1 ha) that is sampled on and off the wellsite – there are four quadrants that collectively comprise the well pad and four quadrants that collectively comprise the reference area.

#### Recovery target

A description of the environmental conditions – expressed in terms of physical, chemical and biological properties – of a site that represents the desired endpoint of successful reclamation. Properly selected reference areas will match the physical, chemical and biological properties describing the recovery target.

#### Reference area (often called a control)

Undisturbed location adjacent to, or nearby, the certified site, from where data are collected for comparison to the certified site data. Each reference area represents the ecological target for the entire certified site, or for a specific portion of the certified site where there is more than one ecological target represented.

Note – the ideal situation would be to have a reference that is of similar age since natural disturbance (i.e., fire) or harvest clearcut, but the reality is that this is unlikely to occur. In lieu of this, we select the adjacent area as representative of what the wellsite would have looked like if it was undisturbed.

#### Site characteristics

Parameters that are used to classify the site during data analysis and reporting.

#### Well bore

The location on the well pad where the well was drilled (and, if produced, where the wellhead was located).

#### Well pad

A subset of the area of land occupied by a wellsite. The well pad is usually a square (approximately 100 m × 100 m) or rectangular area which contains the wellhead and may have contained additional infrastructure for processing the oil or gas.

#### Wellsite

In regulatory language, a wellsite is an area of land leased for the purposes of drilling a well (defined in s. 1(aaaa) of the Environmental Protection and Enhancement Act [7] as: an orifice in the ground that is completed or is being drilled: (i) for the production of oil, oil sands or gas, or (ii) for injection into an underground formation). The wellsite includes the well pad, and may include additional infrastructure such as an access road, a construction material borrow, or an off-site drilling waste sump.

However, for the purposes of the Ecological Recovery Monitoring Program, the term wellsite means the well pad.

#### Wellsite centre

The middle of the well pad. This may or may not be the same as the well bore location.AcronymsABMIAlberta Biodiversity Monitoring InstituteAEMERAAlberta Environmental Monitoring, Reporting and Evaluation AgencyAEPAlberta Environment and ParksAERAlberta Energy RegulatorAITFAlberta Innovates – Technology FuturesANHICAlberta Natural Heritage Information CentreATVAll Terrain VehicleCVCoefficient of VariationCWDCoarse Woody DebrisD&ADrilled and AbandonedDBHDiameter at Breast HeightDNCDid Not CollectDWDDowned Woody DebrisECElectrical ConductivityGISGeographic Information SystemGPSGlobal Positioning SystemSOCSoil Organic CarbonSWDSmall Woody DebrisTNTotal NitrogenUISUnidentified SpeciesVNAVariable Not Applicable

## Footnotes

1 Definitions for key terms used in this document are provided in Section 9

2 Forested Lands for the purposes of the Wellsite Criteria include any treed land, whether or not the forest vegetation is used for commercial purposes. Treed (bush) lands in the White Area that are to be maintained as 'treed' are also considered Forested Land.

3 Definitions for key terms used in this document are provided in Section 9.

6 These are called opportunistic sites.

8 Compositing samples to reduce analysis cost is suggested for measuring TN, SOC, EC and pH.

10 Logs that are completely decomposed in sections, whose outline is discernible but strongly fragmented and whose remaining parts often overgrown are considered as part of the LFH. These are referred to as Class 5 in Fig. 16.

## Footnotes

4 Note the factors and ratings in Table 3 are recommended for consideration – they can be varied to suit alternative priorities.

5 See http://abadata.ca/; note, use of this site requires payment.

11 Only applicable for wellsites certified pre 2004.

7 The person locating the well bore will use the magnetic locator and should not be wearing steel-toed boots as this will interfere with the ability to locate the well bore.

9 In the Pilot Program % cover of seedlings/saplings was estimated. It may be more appropriate in future to count the seedlings/saplings instead. Future repeat sampling could then be used to estimate survival rates.
